# Predicting the Lifetime of Dynamic Networks Experiencing Persistent Random Attacks

**DOI:** 10.1038/srep14286

**Published:** 2015-09-21

**Authors:** Boris Podobnik, Tomislav Lipic, Davor Horvatic, Antonio Majdandzic, Steven R. Bishop, H. Eugene Stanley

**Affiliations:** 1 University of Rijeka, Faculty of Civil Engineering, Rijeka, 51000, Croatia; 2 Boston University, Center for Polymer Studies, Department of Physics, 590 Commonwealth Avenue, Boston, Massachusetts 02215, USA; 3 Zagreb School of Economics and Management, Zagreb, 10000, Croatia; 4 University of Ljubljana, Faculty of Economics, Ljubljana, 1000, Slovenia; 5 Rudjer Boskovic Institute, Centre for Informatics and Computing, Zagreb, 10000, Croatia; 6 University of Zagreb, Physics Department, Zagreb, 10000, Croatia; 7 University College London, Department of Mathematics, Gower Street, London, WC1E 6BT, UK

## Abstract

Estimating the critical points at which complex systems abruptly flip from one state to another is one of the remaining challenges in network science. Due to lack of knowledge about the underlying stochastic processes controlling critical transitions, it is widely considered difficult to determine the location of critical points for real-world networks, and it is even more difficult to predict the time at which these potentially catastrophic failures occur. We analyse a class of decaying dynamic networks experiencing persistent failures in which the magnitude of the overall failure is quantified by the probability that a potentially permanent internal failure will occur. When the fraction of active neighbours is reduced to a critical threshold, cascading failures can trigger a total network failure. For this class of network we find that the time to network failure, which is equivalent to network lifetime, is inversely dependent upon the magnitude of the failure and logarithmically dependent on the threshold. We analyse how permanent failures affect network robustness using network lifetime as a measure. These findings provide new methodological insight into system dynamics and, in particular, of the dynamic processes of networks. We illustrate the network model by selected examples from biology, and social science.

Many real-world complex systems are largely robust to outside attacks and spontaneous fluctuations that cause either temporary or permanent localised breakdowns^1^,^2^,^3^,^4^,^5^,^6^,^7^,^8^,^9^. However, the majority of them ultimately collapse and thus have a finite lifetime. The collapse commonly occurs when the system reaches a critical point and the system abruptly shifts from one phase to another^10^,^11^,^12^,^13^,^14^,^15^,^16^. Examples of real-world complex systems that experience sudden collapse are numerous, e.g., the spread of disease in living organisms^17^, the spread of political dissent in a human society, or the spread of a product sales pattern in economics.

In recent years network science has been utilized to describe complex systems^18^,^19^,^20^,^21^,^22^,^23^,^24^,^25^,^26^,^27^,^28^,^29^,^30^,^31^,^32^, and many dynamic network models have been proposed^33^,^34^,^35^,^36^,^37^,^38^,^39^,^40^ to explain the complicated dynamics that occur in real-world networks. Thus far, most network research has separated the analysis of (a) empirical indicators of critical transitions^11^,^12^,^13^,^14^,^15^,^16^,^41^ from the analysis of (b) network robustness^1^,^2^,^9^,^42^,^43^,^44^,^45^. The studies in group (a) have focused on a phenomenon known as critical slowing down, where the correlations and variance of the fluctuations increase as the system approaches a critical point^14^,^46^. The research in (b) focuses on quantifying vulnerability of different networks under different types of random failures and deliberate attacks.

Most of the existing studies measure the structural robustness of the network by employing the theoretical framework of statistical physics and percolation theory to analyse the impact of a node or link removal on the structural integrity of the network which is commonly assessed by evaluating the size of the giant component. In this regard, network robustness is quantified by the percolation threshold, which is the fraction of the nodes (or links) needed to remove for the network to fall apart (i.e the giant component to vanish).

Recent studies try to estimate percolation thresholds in different types of real networks for the site and bond percolation models^44^,^45^. However, measuring structural network robustness by static percolation thresholds assumes an underlying network with static nodes and links where the failure of a node (or link) is equivalent to a permanent loss of its functionalities. Since in many real-world scenarios the functionality of nodes and links vary over time, theoretical frameworks for quantitative analysis of global, self-healing and targeted recovery processes occurring in different complex networks were also recently proposed^40^,^47^,^48^.

Motivated by the fact that ultimately many real networks collapse and thus have a finite lifetime, here we incorporate both (a) and (b) within a common theoretical framework based on decaying dynamical networks. We focus on networks in which nodes flip between two intrinsic states and also have the ability to control the state of their neighbours. Examples of these networks range from cascading processes in interdependent networks^29^,^30^, epidemic spreading in scale-free networks^17^, dynamic networks able to spontaneously recover^40^ to the glassy dynamics of kinetically constrained models^49^. Among these networks of particular interest is a broad class of complex systems characterized by both a gradually declining trend^37^ and a sudden collapse^50^.

We find that introducing permanent failure in the presence of a stochastic contiguous spreading process produces networks that are continuously decaying only up to a specific, critical point—the network lifetime (*t*_*c*_)—when the network abruptly collapses. We find that this critical point is logarithmically dependent upon the threshold and inversely dependent on the size of the outside attack. In practise when comparing different empirical complex systems, the value of *t*_*c*_ may serve as an indicator of system’s dynamical risk. Using 1/*t*_*c*_ then the larger the parameter value *t*_*c*_, the smaller the dynamical risk.

## Results

Thus far no single network model has been able to explain the process of finite-time decline with the possibility of spontaneous collapse, how ageing or continuous ongoing time-dependent attacks affect network robustness, how long a network can continue to function before it collapses, or how to predict the time of network collapse (*t*_*c*_) prior to its occurrence.

1. The function of a node *n*_*i*_ is dependent upon its nearest neighbours. Generally speaking, a network is robust if its nodes are able to function even when a large fraction of its nearest neighbours have failed. Here this fraction is denoted by *T*_*h*_. If at time *t* the fraction of active neighbours of node *n*_*i*_ is smaller than or equal to *T*_*h*_, then at time *t* + 1 node *n*_*i*_ will become externally inactive with a probability *r*. We use a fractional threshold^51^,^52^, which is more appropriate than the absolute threshold^40^,^53^ when networks have heterogeneous degrees.
2. Each node can internally fail, independently of other nodes, with a probability *p* quantifying the magnitude of the attack. This is related to the static network case when robustness is studied under simultaneous random or targeted attacks^1^,^2^,^9^,^36^,^54^,^55^.
3. Although we assume that a node can recover from an internal failure after a finite period of time (*τ*), internal failure always carries the potential risk of being permanent, an event we define by a probability 1 − *q*. Thus, when a network is influenced by ongoing time-dependent attacks, not every node will be able to recover. A node can be considered active only if it is both internally and externally active^40^. For a more complicated network this will include adding the introduction of link failures or the addition of new nodes (see Methods and Model extensions).

Reference^40^ shows that when the assumptions (i)–(iii) are applied, then when the network model has no permanent failures the local node recoveries (modelled by *τ*) and the stochastic contiguous spreading (modelled by a probability *r*) lead to the spontaneous emergence of macroscopic “phase-flipping” phenomena, corresponding e.g. to flipping between “good” (up phase) and “bad” (down phase) years in economics^51^,^52^. It has also been shown that when the size of the network is increased, the average time spent in the down phase is greater than the average time spent in the up phase. This result has a serious consequence when applied to model world economy and globalization—the model predicts that recession time increases with an increase in the number of countries in the world economy. Reference^51^ shows that when the (i)–(iii) network model has no permanent failures, there are substantial changes in the autocorrelation and variance of fluctuations—characteristic of critical slowing^14^—as the system approaches a critical point.

In seminal studies of the stability of large complex systems, Gardner and Ashby^56^ (numerically) and May^57^ (analytically) examined how such systems maintain stability up to some critical level of connectedness and then suddenly, as the level of connectedness increases, become unstable. In contrast, our focus is on the reverse process in which a continuous decrease in the number of functioning nodes and links between them over time decreases network complexity. We describe the level of network functionality in terms of the fraction of the total number of nodes that are continuing to function^40^ and the fraction of active links, *f*_*l*_^51^. In this dynamical network framework, in contrast to static networks, the network functionality is not only limited to the size of giant component.

The dynamic approach used to quantify the robustness of a network under time-dependent attacks can also be used to address the ageing process of networks. In this case the probability *p* serves as an internal network property^40^. Over time not every internally-failed node can recover, e.g., at the cell level in biology there may be a defective apoptotic process^58^.

**Figure 1:**
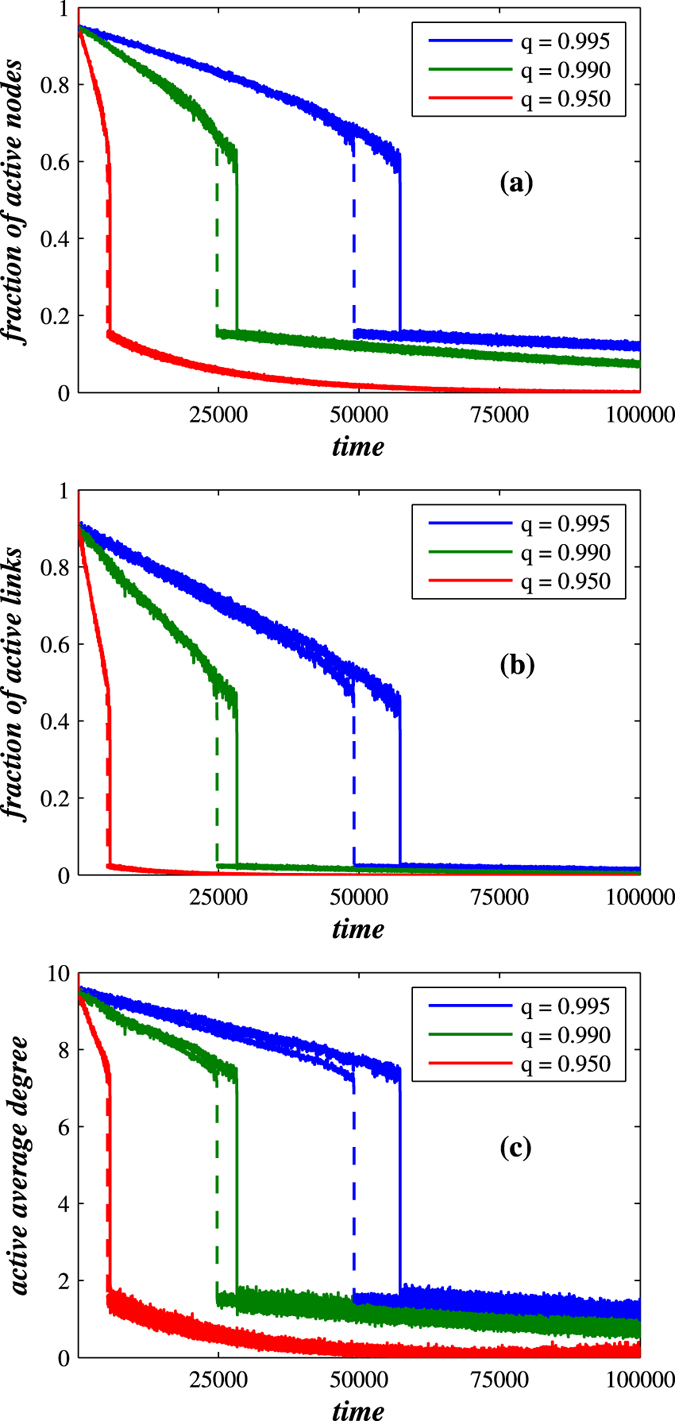
Comparison between BA (solid line) and ER (dashed line) decaying dynamic networks, when 〈*k*〉 = 10. Under the same level of attack, quantified by equal *p*, the BA decaying network exhibits the higher level of robustness than the ER decaying network, where robustness is quantified by larger *t*_*c*_. For (**a**) the fractions of active nodes and (**b**) links and (**c**) the active average degree as a function of time, the BA decaying networks are more robust than the ER networks. By active average degree we mean the average degree calculated for nodes that are active both internally or externally. Here the robustness is defined in a dynamic way—the more robust a network is, the longer it lasts. For both networks we use *p* = 0.001, *r* = 0.8, *T*_*h*_ = 0.5, and *τ* = 50.

To determine how *T*_*h*_ and *q* affect network functionality, we first generate three random Erdős Renyi (ER) networks and three scale-free networks (Barabasi-Albert BA model), each with initial *N*(*t* = 0) = 10,000 nodes and an average degree 〈*k*〉 = 10. Then, each network is placed under persistent attack, quantified by *p*, where we allow nodes in the inactive phase to permanently fail with a probability 1 − *q*. For varying values of *q* for each decaying network, Fig. 1 shows the fraction of active nodes *f*_*a*_, the fraction of active links *f*_*l*_, and the average degree, each as a function of time. In Fig. 1 *T*_*h*_ is fixed but the values of *q* varied, and we see that the network decays continuously until at some specific time (*t*_*c*_) it abruptly and spontaneously collapses; a collapse brought on by a sudden large increase in the number of inactive links and nodes. This sudden collapse does not require any parameter other than *q*. As expected, the more rapid variation of the exponential decay, the sooner the network will collapse. When we compare the fractions of active nodes and links we see that, when the network collapse occurs, the fraction of active links decreases substantially more than the fraction of active nodes, where the fraction of active links is approximately 1 − *r*.

Our analysis addresses both how long a dynamic network will function before collapse, and how robust it will be under long-term continuous attack, i.e., the larger the network lifetime *t*_*c*_, the more robust the network. This study of robustness of dynamic networks under continuous attack is highly relevant to both researchers and practitioners. For example, it can be used in military science for determining how a military network can remain robust under persistent enemy attack, or a goal in finance is determining how a financial system can remain robust when a fraction of its banks fails over time. References^1^,^2^ reported that scale-free networks are more robust than ER networks to multiple random and simultaneous attacks.

We next compare the robustness of decaying BA and ER networks, and our definition of network robustness is dynamic, i.e., the more robust a network is, the longer it will last. Figure 1 compares the robustness of decaying BA and ER networks under continuous long-term attack, measured by *p*, as a function of time. We use the same parameters for both types of network. Using network lifetime to quantify dynamic network robustness, Fig. 1 shows that, even when nodes and links fail, BA networks are generally more robust than ER networks. A dynamic BA network is typically able to survive longer with higher values of *t*_*c*_ than an ER network before it exhibits an abrupt drop in the fraction of its active nodes and links and its average degree. Figure 1(c) shows that, for the *q* values displayed, the average degree of a dynamic BA network lasts longer (i.e., *t*_*c*_ is larger) than the average degree of a dynamic ER network.

**Figure 2:**
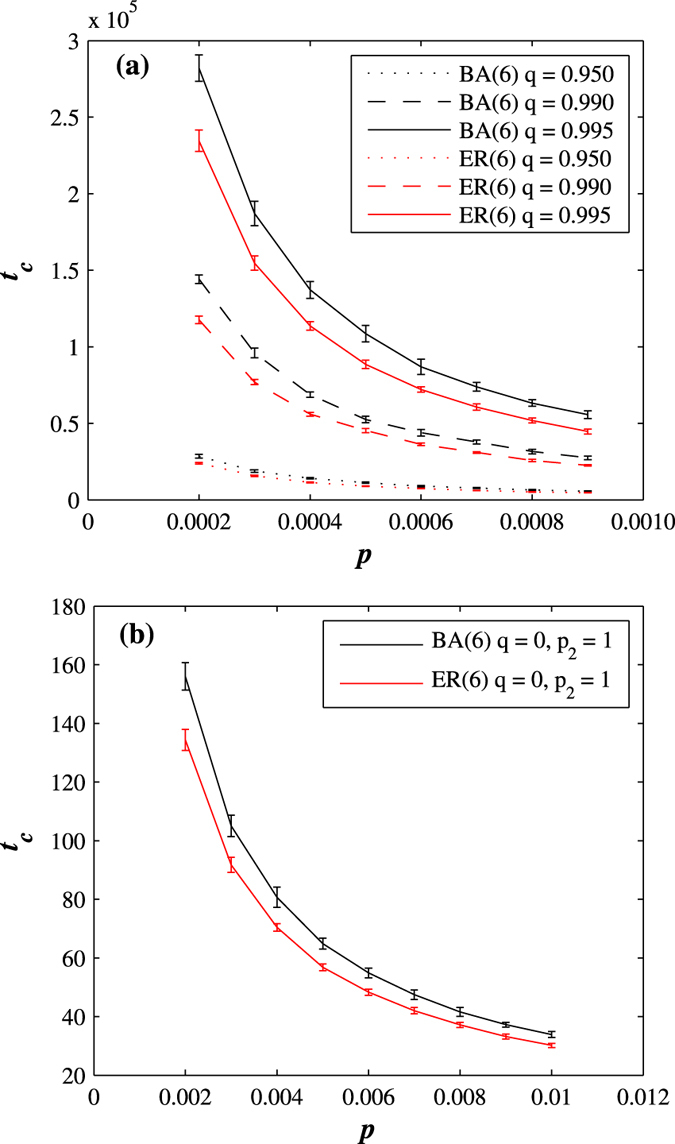
Robustness of BA and ER decaying networks for different values of *q*, where *t*_*c*_ serves as the measure of robustness. We show *t*_*c*_ versus *p* for (**a**) *q* = 0.995, 0.99, 0.95. The BA decaying networks exhibit higher level of robustness than the ER decaying networks. We use *r* = 0.8 and 〈*k*〉 = 6. (**b**) For BA and ER decaying dynamic networks when a node cannot recover (*q* = 0), for 〈*k*〉 = 6 the larger time *t*_*c*_ (the larger robustness) we obtain for BA decaying network than for ER decaying network. Here, the larger the time *t*_*c*_, the longer the network lifetime, and the more robust the network.

**Figure 3:**
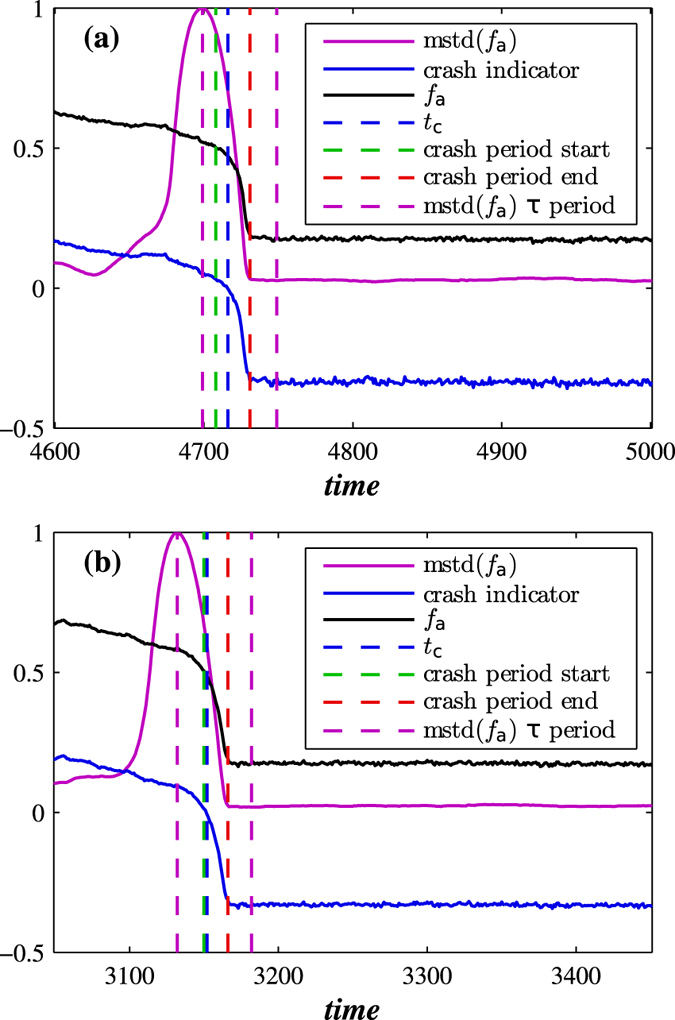
Indicators of network crash. Shown are two indicators together with the fraction of active nodes *f*_*a*_ obtained for (**a**) BA and (**b**) ER decaying network when *q* = 0.99 and 〈*k*〉 = 6. We show indicator I (mstd), moving standard deviation of fraction of active nodes (forward method) with window size *τ* = 50 (the same *τ* as in recovery process), and indicator II (crash indicator), the average fraction of active neighbours in excess of threshold *T*_*h*_. When the average fraction of active neighbouring nodes drops below the threshold *T*_*h*_ for the first time (*t*_*c*_), cascading failures trigger a network crash. We use *p* = 0.003, *T*_*h*_ = 0.5, and *r* = 0.8.

To determine whether this difference in robustness is general or sample-dependent, we compare decaying BA and ER networks for different *q* values. For fixed *T*_*h*_ and *r*, and varying values of *q*, Fig. 2(a) shows the relationship between *t*_*c*_, whose precise definition will be explained in Fig. 3, and *p*. The BA decaying networks generally exhibit a higher level of robustness than the ER decaying networks^1^. We find a similar dependence between *t*_*c*_ and *p* for varying network degrees.

The dynamic approach taken here also allows the non-trivial possibility that, following an attack, nodes can remain permanently damaged, corresponding to the *q* = 0 case in which no recovery is allowed. This allows us to compare our results with studies in which robustness is analysed in static networks under either simultaneous random or targeted attack^1^,^2^,^9^,^36^,^54^,^55^. Note that when *q* = 0, the period *τ* becomes irrelevant. For a fixed *T*_*h*_ = 0.5 and 〈*k*〉 = 6, Fig. 2(b) shows how the time *t*_*c*_ is affected by the level of outside attack, quantified by *p*. Again as in Fig. 2(a), the larger the level of outside attack, the smaller the network lifetime. Because no recovery is allowed for this case, the lifetime values in Fig. 2(b) are much smaller than those in Fig. 2(a). When *q* = 0, decaying BA networks exhibit a significantly higher level of robustness than decaying ER networks, and the times *t*_*c*_ calculated for BA networks are consistently larger than those calculated for ER networks—the dependence between *t*_*c*_ and *p* follows a hyperbolic function.

In a complex dynamic system, thresholds and critical points control the transition process between different states^59^. A phase transition occurs when a system reaches a system threshold. Using this perspective, the network decline and collapse numerically described above can also be treated analytically. Note that at time *t* − *τ*, attacks on a network that has *N*(*t* − *τ*) active and inactive living nodes will cause *pN*(*t* − *τ*) nodes to switch to the internally inactive phase. The change in the number of living, or active, nodes is proportional to the probability that a node that internally failed at *t* − *τ* will not recover, 1 − *q*, thus


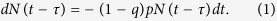


The number of living nodes remaining is determined by an exponential decay *N*(*t*) = *N*(0)exp(−(1 − *q*)*pt*) with a decay rate of *λ* = (1 − *q*)*p*, where *N*(*t*)/*N*(0) is the average fraction of living nodes equivalent to the average fraction of living neighbours of each node. For simplicity, let us assume that all nodes have the same initial degree *k*, remembering that node *n*_*i*_ will be active if the number of its active neighbours is larger than *m* = *T*_*h*_*k*. For a network with degree distribution *f*_*k*_ at time *t*, the fraction of 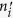
s failed neighbouring nodes *N*_*f*_ (*t*)/*N*(*t*) among *N*(*t*) living nodes previously estimated as *k*(*t*) = *k* exp(−(1 − *q*)*pt*), can be approximated using the probabilities of internal and external failures, *p* and *E*[*m*, *k*(*t*)],


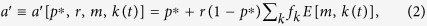


where 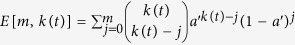 and p^*^ = 1 − exp(−*pτ*)^40^. In the above figures we consider the ratio between the living active and the initial number of nodes *N*_*a*_(*t*)/*N*(0), which is equivalent to


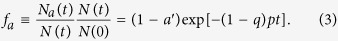


Here exp[−(1 − *q*)*pt*] is due to the continuous decline of the network, and 1 − *a*′ indicates the sudden network crash occurring when the fraction of living nodes *k*(*t*)/*k* approaches the threshold *T*_*h*_ = *m*/*k*. At this limit, from Eqs. (2) and (3) we see that *E*[*m*, *k*(*t*)] → 1 and the probability of external failure dominates *p*.

Although predictive power is important in any scientific endeavour, it is widely assumed that predicting critical transitions is extremely difficult because any change in the dynamic state of a system immediately prior to reaching the critical point will be slight^11^. Unlike early-warning indicators that utilize recent system outputs to detect impending system collapse, we use past data to estimate network parameters, from which we generate numerical simulations that describe the network state at any future time, including the time of network failure *t*_*c*_. Thus we are able to accurately predict the time of network failure.

Figure 3 shows the predictive power of this dynamic approach when applied to both ER and BA networks which are in the process of decaying. It shows the fraction of active nodes for time scales that include the network crash and also two important values: (I) the standard deviation of the fraction of active nodes calculated using moving boxes similar to the approach proposed in Reference^11^ and (II) the average fraction of active neighbours—active both internally and externally—above the threshold *T*_*h*_. Equations (2) and (3) provide a theoretical explanation for II, predicting that when the fraction of currently living nodes approaches the threshold that controls the external failures, the probability of critical transition increases and cascading failures trigger a network crash. Note that just prior to a network crash, indicator I increases substantially and thus is an indicator of the impending failure and a predictor of the time *t*_*c*_, i.e., it predicts when network failure will occur. Reference^11^ suggests that as a dynamic system approaches a critical threshold its state at any given moment increasingly resembles its previous state, implying an increase in variance and autocorrelation as reported for dynamic networks in Reference^51^. We find that this predictive power holds for both BA and ER decaying networks for a wide variety of parameters. In Fig. 3 the values of II shift from positive to negative and the time when this occurs can also be used as a predictor of the time of network collapse *t*_*c*_. In addition to the two numerical indicators, we also suggest an analytic formula for *t*_*c*_, obtained by equating (1 − *a*′)exp[−(1 − *q*)*pt*] in Eq. (3) with *T*_*h*_, so that


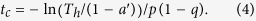


**Figure 4:**
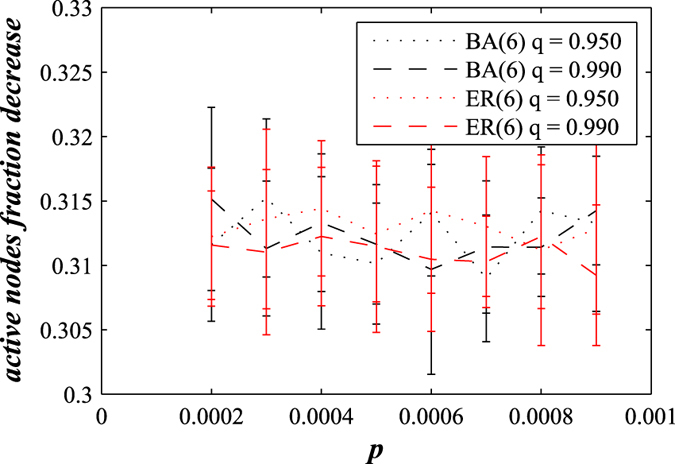
For the varying *q* values, for both decaying ER and decaying BA network we show the size of the decrease of *f*_*a*_ during the network crash, equal to *T*_*h*_ − *r*.

In the decaying network approach, the proportional threshold *T*_*h*_ controls both node-level crashes and “macro” network-level crashes, which makes *T*_*h*_ a breakdown threshold. For a general set of network parameters, the time of network failure is the ratio between the closeness of the fraction of active nodes to the threshold, −ln(*T*_*h*_), and the rapidity of their approach to the threshold, *p*(1 − *q*). In Fig. 3(a,b) we see that *t*_*c*_ typically corresponds to the beginning of the network failure period. Additionally, for varying values of *q* in Fig. 4 we find that the fraction of active nodes decreases during a network failure in both decaying BA and ER networks for *T*_*h*_ − *r*.

**Figure 5:**
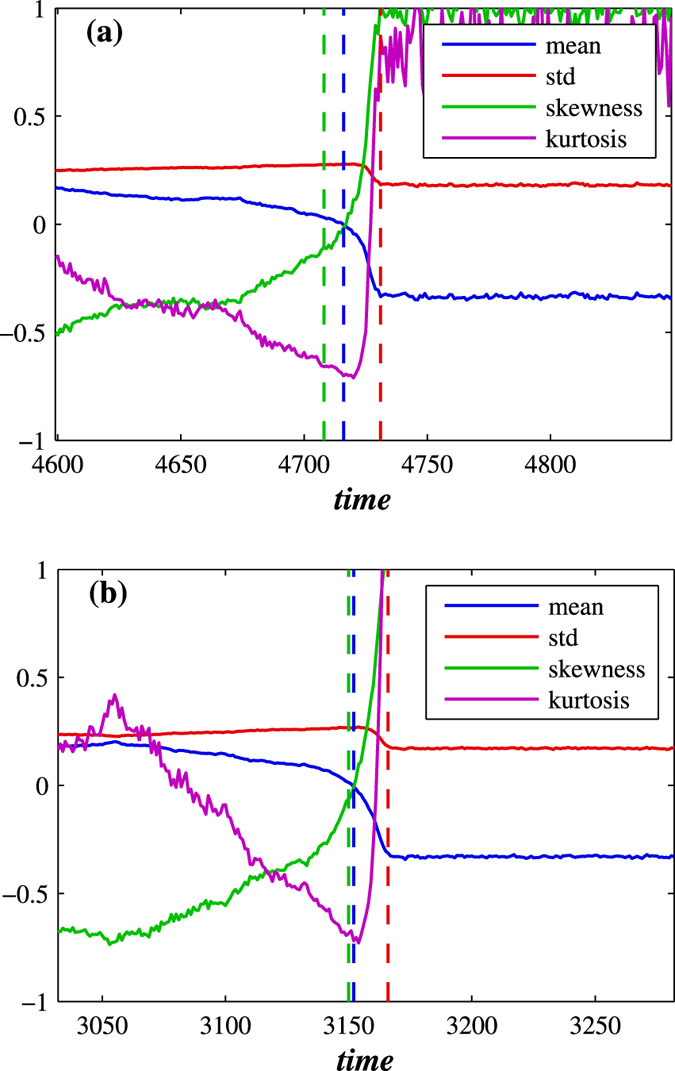
Statistics of the fraction of active neighbours in excess of threshold *T*_*h*_ for (**a**) decaying BA and (**b**) decaying ER network with 〈*k*〉 = 6. We show how the first four moments change over time just before, during, and just after the crash. Just before the network crash, skewness and kurtosis dramatically and abruptly increase.

**Figure 6:**
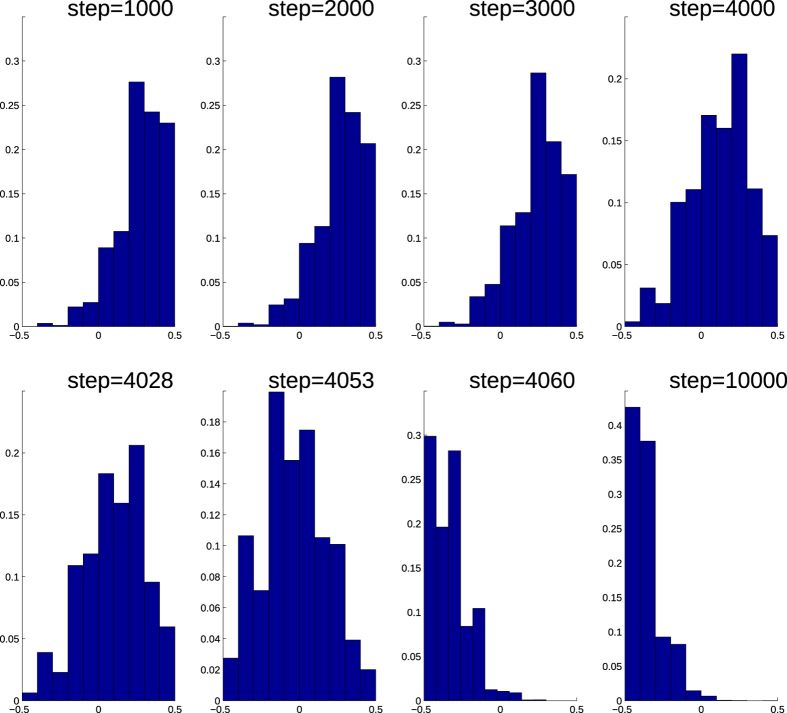
Distribution of the fraction of active neighbours in excess of threshold *T*_*h*_ substantially changes during the network failure.

Figures 1 through 4 describe how *t*_*c*_ is affected by *T*_*h*_, *p*, and *q*. Figure 3(a,b) show that during a network crash the fraction of active neighbouring nodes approaches the threshold. We also find that the time series of the fraction of active neighbours of each node above the threshold *T*_*h*_ substantially changes over time. Figure 5(a,b) display the average representing our indicator II, calculated for each network in Fig. 3(a,b), and also the higher moments, variance, skewness, and kurtosis. For different times prior, during, and after the network failure, Fig. 6 shows the distribution of the fraction of active neighbours of each node above the threshold *T*_*h*_. Just prior to network failure, skewness, kurtosis, and the distribution substantially change.

In estimating the time of a future network crash, we calculate network parameters from its creation at time *t* = 0 to some time *t* during which the fraction of active nodes *f*_*a*_ decreases from 1 to some value *f*′. When we numerically generate the network we thus record the time *t* at which *f*_*a*_ reaches *f*′. Then the true prediction of future network collapse is not *t*_*c*_ but *t*_*c*_ − *t*, i.e., *t*_*c*_ represents the entire lifetime of the network, and our interest is in estimating the lifetime remaining after *t*.

### Decay with the Stochastic Threshold

It is known in the field of finance that increasing the number of links between banks reduces the systemic risk and the risk of financial contagion^60^. Until now we assumed that the threshold *T*_*h*_ quantifying network robustness was the same for each node. Such assumption is not appropriate in the real-world of finance because not every bank is equally robust. We therefore assume that the robustness of each node is locally defined. For example, suppose two banks, X and Y, have an equal debt of 10B$ but different assets of 15B$ and 12B$, respectively, and each allocates 3B$ to three neighbouring banks. If the criterion of bank solvency is that assets must be larger than debts, X can survive even when all neighbouring banks fail, but Y cannot survive if more than one neighbouring bank fails. Therefore X is more robust than Y.

**Figure 7:**
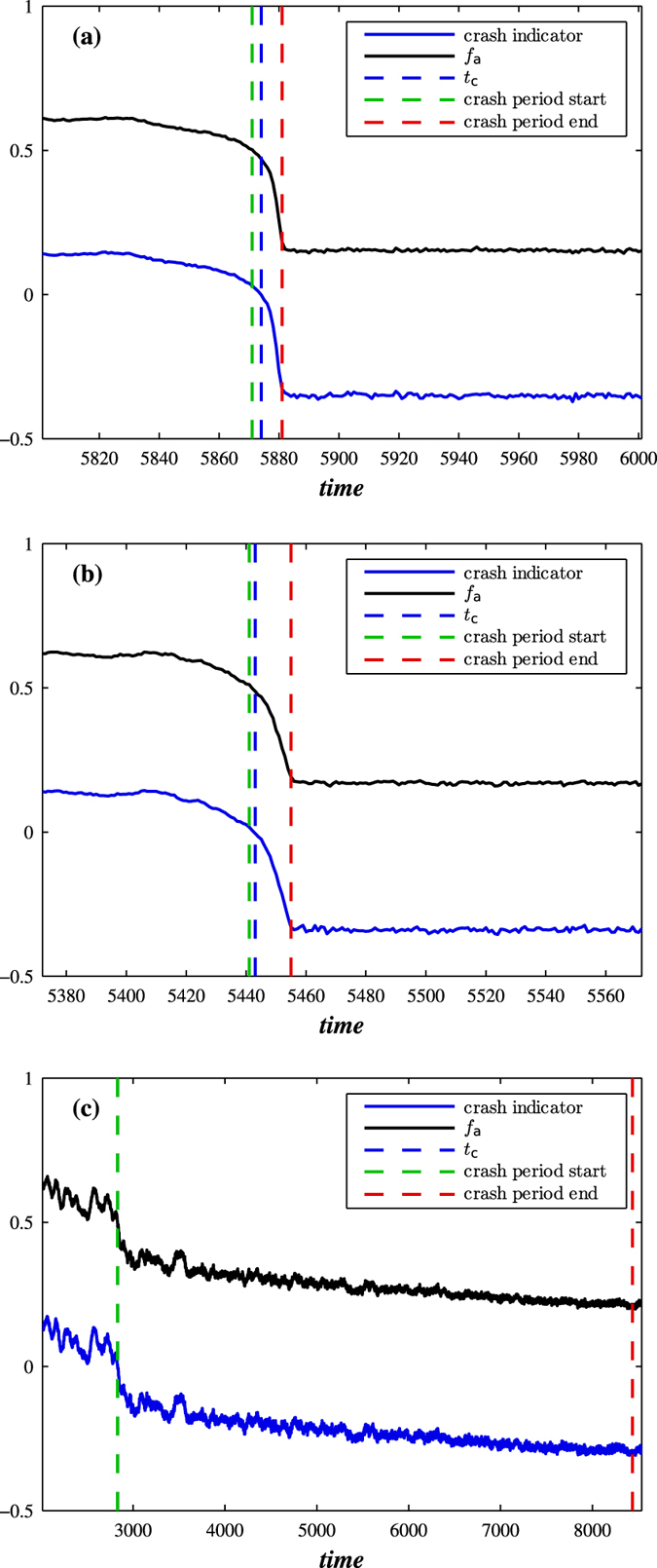
The effect of varying threshold *T*_*h*_ on a network crash. Shown are the fraction of active nodes *f*_*a*_ and indicator II (crash indicator), the average fraction of active neighbours in excess of threshold *T*_*h*_. We use the BA network with 〈*k*〉 = 10 and *p* = 0.003, *r* = 0.8, *q* = 0.99, and the varying threshold *T*_*h*_ taken from a Gaussian distribution G(*μ* = 0.5, *σ*) where (**a**) *σ* = 0, (**b**) *σ* = 0.1, and (**c**) *σ* = 0.2.

We next assume that the robustness of each node is randomly chosen from a Gaussian distribution with a mean 0.5 and a varying standard deviation. Figure 7 shows standard deviations equal to (a) 0, (b) 0.1 and (c) 0.2. In all three cases we find that the estimate for *t*_*c*_ of Eq. (4), where now *T*_*h*_ corresponds to the mean of the Gaussian, correctly predicts the time of network breakdown. However we find that the standard deviation of the fraction of active nodes rapidly changes as the uncertainty of the threshold increases. In the smaller *T*_*h*_ uncertainty shown in Fig. 7(a,b), the standard deviation of the fraction of active nodes is characterized by one unique large bump. In contrast, when the standard deviation in *T*_*h*_ changes from 0 to 0.2, we find that the unique bump disappears and is replaced by many smaller flash fluctuations, because different nodes in the network are characterized by different thresholds and thus do not collapse simultaneously. We conclude that heterogeneity in node robustness may create a more robust network, and allows us to devise a strategy for preventing a partially dissolving network from collapsing completely.

Note that Fig. 7 suggests that the average fraction of active neighbours in excess of threshold *T*_*h*_ slowly decreases over time, with a functional dependence approximated by a linear dependence, *I*_0_ − *αt*, where each node *i* has its own threshold *T*_*hi*_. *α* represents a slope of the linear dependence that is comparable to *p*(1 − *q*) [see Eq. (3)], and *I*_0_ is intercept. Each node collapses when approximately *I*_0_ − *αt* = *T*_*hi*_. Then, for two nodes having thresholds *T*_*h*1_ and *T*_*h*2_, we obtain


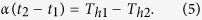


The more robust the node, the longer it survives. The larger the uncertainty in *T*_*h*_, the larger the difference in the time of collapse. If *T*_*h*1_ = *T*_*h*2_ as in Fig. 7(a), we obtain a virtually simultaneous collapse of all nodes. We may speculate that if *t*_2_ − *t*_1_ is larger than the average relaxation time during the cascade process when *T*_*h*_ is homogeneous, there will be no unique large bump in the standard deviation of the fraction of active nodes, but two separate smaller bumps as in Fig. 7(c). In Fig. 7 we also find that when the standard deviation of *T*_*h*_ increases, the relaxation time of the cascade process also increases.

**Figure 8:**
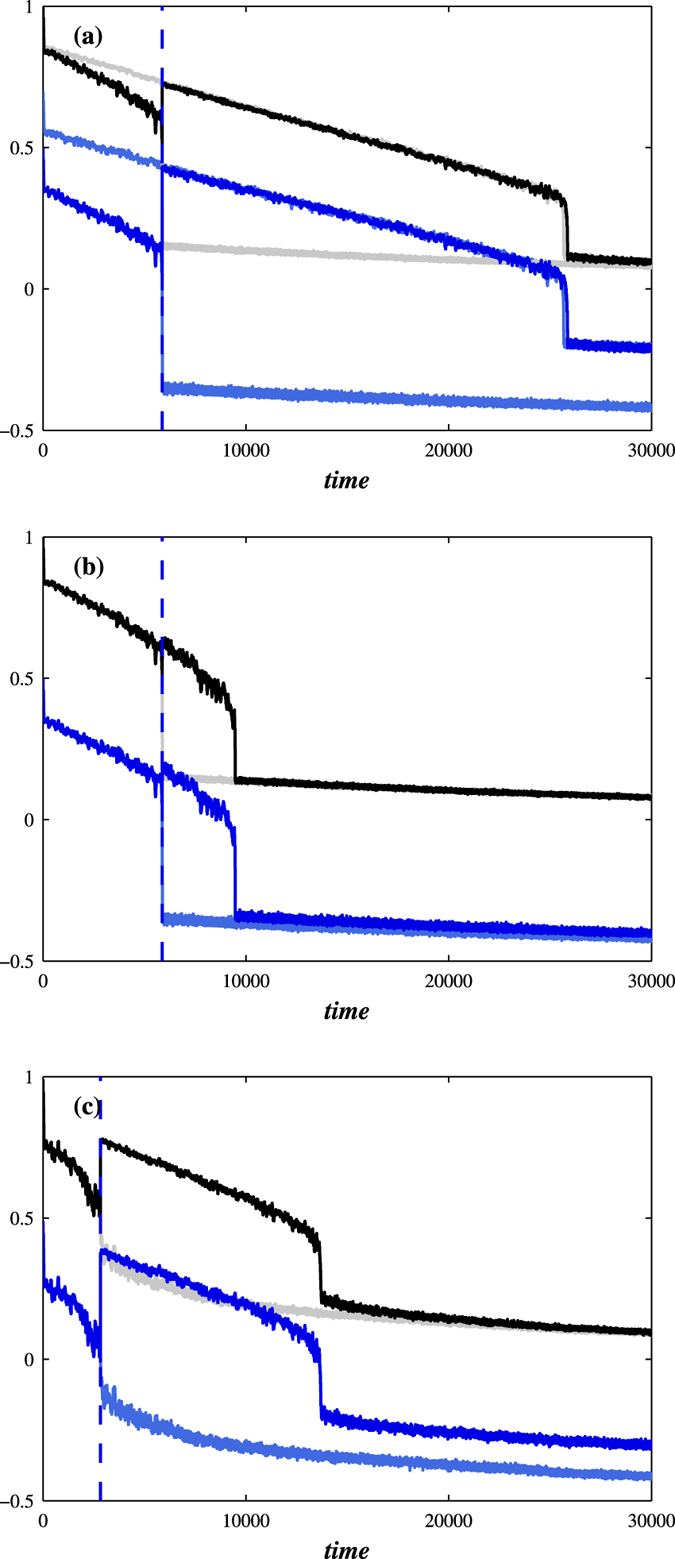
By decreasing the threshold *T*_*h*_, and thus increasing the robustness just before the network collapse, the fraction of active nodes rises abruptly, and the network becomes more stable. Shown are the fraction of active nodes (black) and crash indicator (blue) for the BA network with 〈*k*〉 = 10 where at *t*_*c*_ the constant threshold *T*_*h*_ is decreased by 0.2 (**a**) for each node and (**b**) only for the 1,000 nodes with the largest degree. (**c**) For the same network, but with a varying threshold taken from a Gaussian distribution G(*μ* = 0.5, *σ* = 0.2), the treatment of decreasing the threshold *T*_*h*_ by 0.2 for nodes which *T*_*h*_ is greater than 0.5 is made at *t* = *t*_*c*_. We use *p* = 0.003 and *r* = 0.8. Light colours indicate the fraction of active nodes and crash indicator without the treatment.

When applying forecasting theories in practice, the effort of the modelling procedure must be to approximate the empirical system. Often we must also assume that the model parameters, commonly obtained by fitting past data, do not change during a period of forecasting. Thus, if the financial system of a country, modelled by a set of parameters, approaches a financial breakdown and the government intervenes, the model parameters must be changed. To this end, in Fig. 8 the average fraction of active neighbours in excess of threshold *T*_*h*_ slowly decreases over time and just before the network collapse at *t* = *t*_*c*_ the government intervenes by decreasing the threshold *T*_*h*_ to all nodes causing an abrupt jump in the fraction of active nodes. As one may expect this government measure just postpones the network collapse for some future time as seen in Fig. 8(a). Clearly this modelling procedure seems reasonable for countries where the policy of constant increase in indebtedness eventually must lead to a bankruptcy. Different government policies are shown in Fig. 8(b,c). In Fig. 8(b) the government, following “too big to fail policy”, intervenes at *t* = *t*_*c*_ by decreasing the threshold *T*_*h*_ for only 100 largest nodes. Similarly in Fig. 8(c) this time when the threshold is a stochastic variable taken from a Gaussian *N*(0.5, *σ* = 0.2) at *t* = *t*_*c*_ the government decreases the threshold *T*_*h*_ for only nodes with threshold larger than 0.5 (the less robust nodes).

### Characterizing *t*_*c*_ dependence on real network structure

As the robustness of a network largely depends on the network’s structure, in addition to the analysis of ER and BA random network models, we also analyse dynamics of the decaying dynamic network model on a variety of real networks topologies. We consider a collection of 24 real-world networks of technical (man-made), biological and social systems with different structural properties (e.g. number of nodes, degree distributions, absorptivity, clustering coefficient and diameter) in order to show how they characterize dynamical robustness measure *t*_*c*_ of the model.

For technical systems representatives we include physical infrastructure networks (US Power Grid^61^, Euro Road^62^, Open Flights^63^ and US Airports^63^), and computer networks^64^ that include networks of autonomous systems of the Internet from CAIDA and Route Views projects (AS-733, Oregon1, Oregon2) together with peer-to-peer file sharing network Gnutella. Protein-protein interaction maps for the human^65^, plant^66^, worm^61^ and yeast^67^ interactome network from CCSB Intercome Database are chosen as representatives for biological systems. Finally, social systems representatives include scientific collaboration networks^68^ (CondMat, HepTh, NetScience), interaction networks in online social networking platforms^69^ (ego-Facebook, ego-Twitter, ego-gPlus) and personal communication networks (PGP^70^ and email URV^71^), political blogosphere (Blogs^72^) and connections between board of directors in public companies in Norway (Boards^73^).

In Supplementary Table S1 we provide the complete list of the analysed real networks with their structural properties. All networks were acquired from a variety of online freely available data sources specified also in the table. All originally weighted and/or directed networks were reduced to their unweighted and undirected projections without multiple links. Additionally, for networks with self-loops we analyse their structures with and without self-loops, while for disconnected networks, we also analyse their giant components resulting the analysis of overall 38 topological structures with different structural properties.

For all of these topological structures we report the averaged *t*_*c*_ over 20 realizations of the model dynamics with *p* = 0.003, *q* = 0.99, *r* = 0.8 and *T*_*h*_ = 0.5 in Supplementary Table S2. Supplementary Figure S3–S12. show a realization of the chosen model dynamics of the fraction of active nodes for which *t*_*c*_ in the table was calculated. In order to easier compare *t*_*c*_ results for the set of real networks with varying structural properties, in Supplementary Figure S1. For the same model we report, the *t*_*c*_ dependence on the average degree of the two BA random networks with different size. Similarly, in Supplementary Figure S2, for BA random network with tunable clustering^74^ we report how *t*_*c*_ depends on average clustering coefficient and transitivity.

**Figure 9:**
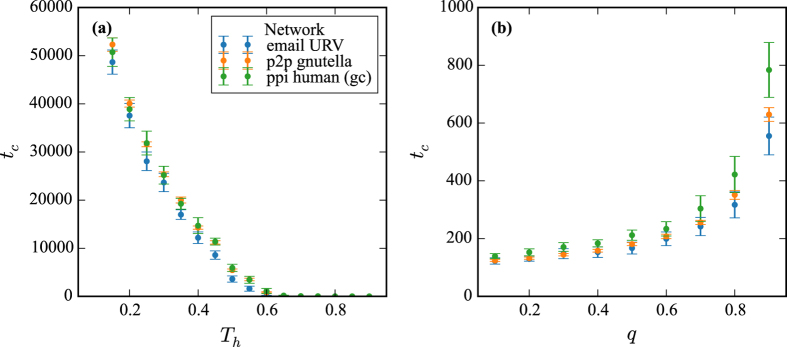
The dependence of *t*_*c*_ on (**a**) *T*_*h*_ and (**b**) *q* for three selected real networks. We use model with fixed parameters *p* = 0.003 and *r* = 0.8, and in (**a**) *q* = 0.99 while in (**b**) *T*_*h*_ = 0.5. The dependence is in agreement with the results of Eq. (4).

For three selected real networks (email personal communication network at URV, peer-to-peer file sharing network Gnutella and the human protein-protein interactome network) in Fig. 9 we show the dependence of *t*_*c*_ on (a) *T*_*h*_ and (b) *q*. The dependence is in agreement with the results of Eq. (4).

## Discussion

In both the natural and social sciences a wide range of real-world complex networks have a finite lifetime characterized by abrupt shifts between phases. One of the differences between the behaviour of natural systems and social systems near a critical point is that the sudden random change commonly occurs at a fixed point in natural systems^75^. In contrast, in social sciences even a critical point is a random variable and, although there is an expectation that some radical change is imminently probable, it is generally assumed that it is not possible to predict the exact time of its occurrence. We have explored the predictive power for a particular class of dynamic non-equilibrium decaying networks. In our network model the proportional threshold *T*_*h*_ responsible for node-level crashes is the parameter that also controls “macro” network-level crashes. Our dynamic network study includes cases in which nodes can recover (*q* ≠ 0) or remain in a failed state (*q* = 0) after a failure caused by the ageing process or by severe hostile time-dependent attacks. Model extensions are included in the Methods section. How successful our network model might be when applied in practice depends first on how capable we are in estimating model parameters, especially the critical threshold^76^ and second, how good our network is as a proxy for a real-world complex system. In practice the second issue is common when real-world stochastic systems, composed of large number of units, such as financial systems, are modelled using such theoretical stochastic processes as multivariate autoregression. In addition, real-world networks are not isolated, but interact with other networks^29^. Due to interdependencies between different networks, a shock to a particular network may change both its own parameters and the parameters of all the other networks in the system. Because the time to the network breakdown is strongly dependent upon network parameters, it is clear that any change in network parameters will also affect our prediction of the timing of network collapse.

## Methods and Model Extensions

### Adding new nodes

**Figure 10:**
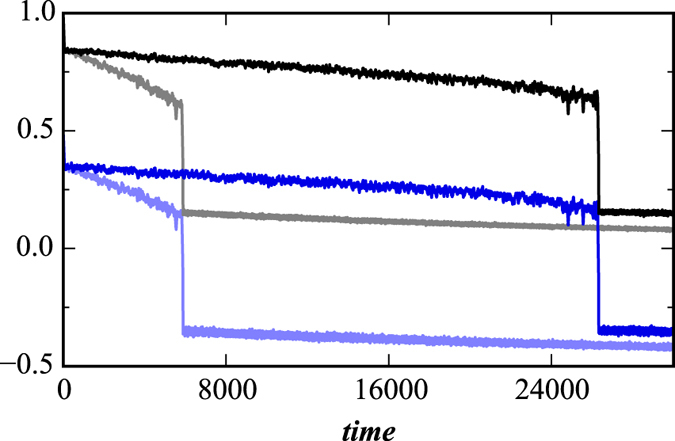
Allowing that new nodes are added while the network decays, when the average fraction of active neighbours in excess of threshold *T*_*h*_ reaches zero, the network abruptly drops. We use the BA network with 〈*k*〉 = 10 and *p* = 0.003, *r* = 0.8, *p*′ = 0.00002, and the constant threshold *T*_*h*_ = 0.5. Shown are the fraction of active nodes (black) and crash indicator (blue). Light colours indicate the fraction of active nodes and crash indicator without adding new nodes.

The model for network growth can be extended such that nodes can be created as well as destroyed. We assume that, at each time *t*, among *N*(*t*) living nodes, each living node can create a new node with a probability *p*′. Figure 10 shows that the average fraction of active nodes suddenly drops when the average fraction of active neighbours in excess of threshold *T*_*h*_ reaches zero.

### The phase-flipping with decay

**Figure 11:**
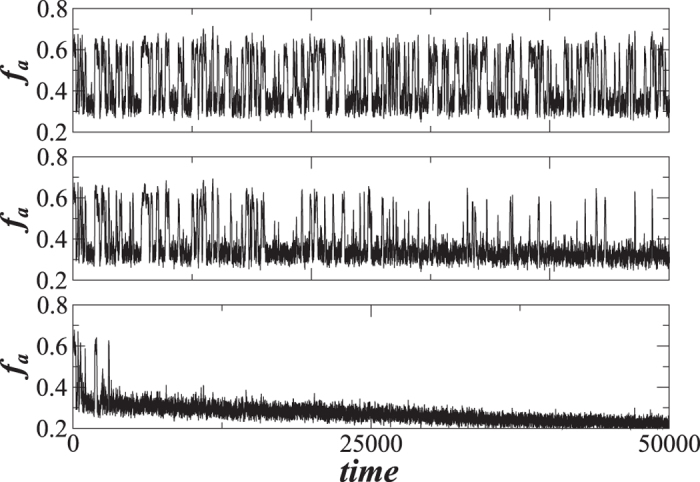
Phase-flipping with decay mechanism for the decaying BA network with parameters *T*_*h*_ = 0.50, *τ* = 50, the average degree 〈*k*〉 = 3, 1000 nodes. With decreasing *q*, from 0.999995, 0.9999, and 0.999 (from top to bottom) we show that the average *f*_*a*_ decays with time where the smaller *q*, the faster the decay.

Reference^40^ reports that introducing both dynamic recovery and stochastic contiguous spreading leads to spontaneous collective network phase-flipping phenomena which critical behaviour of the system is characterized by first-order phase transition and hysteresis. For the *q* = 1 case, we set the network parameter to enable phase-flipping between two stable states. Figure 11 shows that when the decay mechanism is included (*q* ≠ 1) with a decreasing parameter *q*, the fraction of living nodes gradually disappears over time and also the occurrence of phase-flipping phenomena and the collective network mode.

### Introducing link failures

We assume that in decaying networks both links and nodes can fail at any time *t*. We also assume that any link 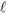 can independently (internally) fail with a probability 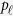. We define a link to be “healthy” (active) if it is active both internally, controlled by 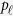, and externally, controlled by *p* in (ii). Defining a dynamic network includes determining which part of the parameter space is characteristic of a stable network regime and which is characteristic of an unstable network regime. Our dynamic network is defined by the three probability parameters, *p*, *r*, and 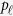. We provide analytical results for the hysteresis behaviour in parameter space for the case when nodes always recover after an inactive period (*q* = 1), here enclosed by manifolds comprising spinodals that separate the regions of stability and instability. For an active node *i* with *k*_*i*_ neighbours, the link between *n*_*i*_ and another node *n*_*j*_ thus fails either due to an internal link failure (*X*) with probability 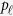 or an external node failure *n*_*j*_ (*Y*) equal to *a*—in the latter case and according to definition, when *n*_*j*_ fails, all of its links have failed. Thus, assuming *X* and *Y* are independent, the link is active with probability 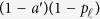 and accordingly inactive with probability 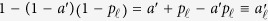. Similar to the case in (i), when the links fail internally we define node *n*_*i*_ to be active only when there are more than *m* active links—not nodes, as in (i). Thus by


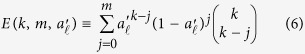


we define the probability that the neighbourhood of node *n*_*i*_ is critically damaged with more than *m* broken links. Finally, we derive the probability that a randomly chosen node *n*_*i*_ with degree *k* is inactive, which is equal to the fraction of inactive nodes,


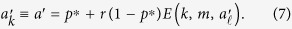


**Figure 12:**
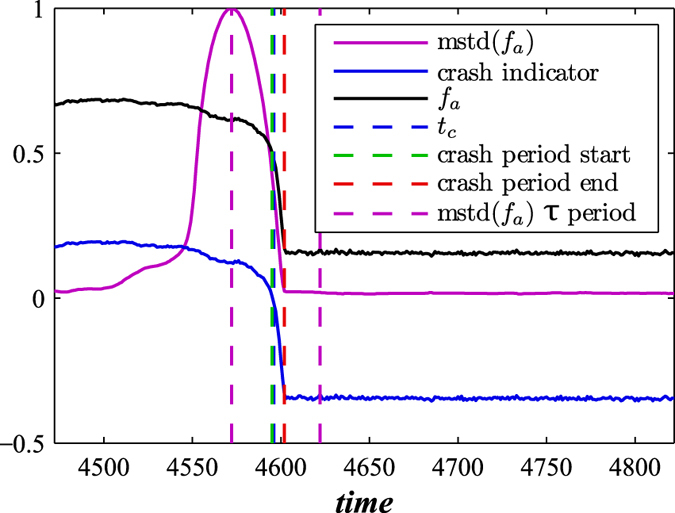
Indicators for network crash, similar as in Fig. 4 but now we include internal link failures. Shown are 3 indicators obtained for an ER decaying network when *q* = 0.99, *T*_*h*_ = 0.99, *r* = 0.8, *p* = 0.003, and 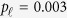.

Here we apply the probability formula *P*(*X* ∪ *Y*) = *P*(*X*) + *P*(*Y*) − *P*(*X*)*P*(*Y*) for two independent events. Note that this equation is derived under the assumption that internal failures affect external failures and thus the notion that external and internal failures are independent is only approximately true. Figure 12 presents an ER decaying network and shows the predictive power for the case in which internal link failures are taken into consideration.

**Figure 13:**
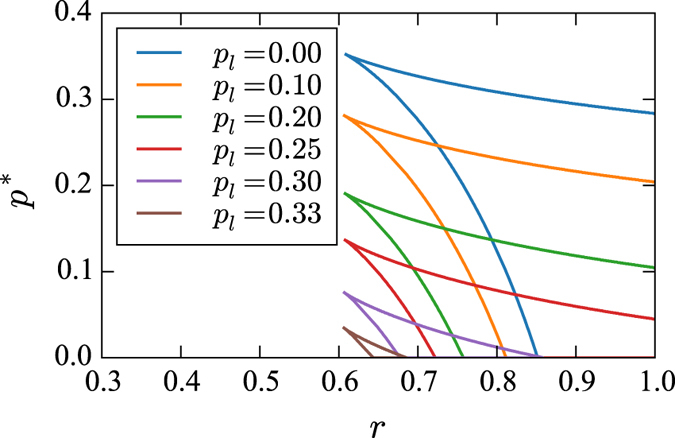
For *q* = 1 case, we show the phase diagram with outline of the hysteresis region in model parameter space (p^*^, *r*) for fixed values of 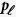 obtained from Eq. (7)**, where** p^*^ = 1 − exp(−***pτ***). Hysteresis region is bounded with two spinodal lines merging at a critical point. We use *k* = 10 and *m* = 4.

Equation (7) assumes that all nodes have the same degree, and thus for simplicity we here analyse random networks that are regular. Figure 13 shows the mean-field (MFT) prediction for the position of spinodals (merging at a critical point) in parameter space (p^*^, *r*) for fixed values of 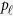 obtained from Eq. (7), where p^*^ = 1 − exp(−*pτ*) 40. In parameter space, the hysteresis region is enclosed by comprising spinodals.

## Additional Information

**How to cite this article**: Podobnik, B. *et al.* Predicting the Lifetime of Dynamic Networks Experiencing Persistent Random Attacks. *Sci. Rep.* **5**, 14286; doi: 10.1038/srep14286 (2015).

## Supplementary Material

Supplementary Information
