# Supplementary material for: Predicting the Lifetime of Dynamic Networks Experiencing Persistent Random Attacks
Source: Sci Rep. 2015 Sep 21;5:14286. doi: 10.1038/srep14286 (PMC4585692; doi:10.1038/srep14286)
Supplement: Supplementary Information [file srep14286-s1.pdf]

Supplementary information for “Predicting the Lifetime of Dynamic Networks Experiencing Persistent Random Attacks”

Boris Podobnik[1,2,3,4,*], Tomislav Lipic[2,5], Davor Horvatic[6], Antonio Majdandzic[2,+], Steven M. Bishop[7,+], H. Eugene Stanley[2,+]

[1]University of Rijeka, Faculty of Civil Engineering, Rijeka, 51000, Croatia
[2]Boston University, Center for Polymer Studies, Department of Physics, 590 Commonwealth Avenue, Boston, Massachusetts 02215, USA
[3]Zagreb School of Economics and Management, Zagreb, 10000, Croatia
[4]University of Ljubljana, Faculty of Economics, Ljubljana, 1000, Slovenia
[5]Rudjer Boskovic Institute, Centre for Informatics and Computing, Zagreb, 10000, Croatia
[6]University of Zagreb, Physics Department, Zagreb, 10000, Croatia
[7]University College London, Department of Mathematics, Gower Street, London, WC1E 6BT, UK
[*]Email: bp@phy.hr
[+]these authors contributed equally to this work

Contents

S1 Supplementary figures **2**
S2 Supplementary tables **9**
References **11**

S1 Supplementary figures

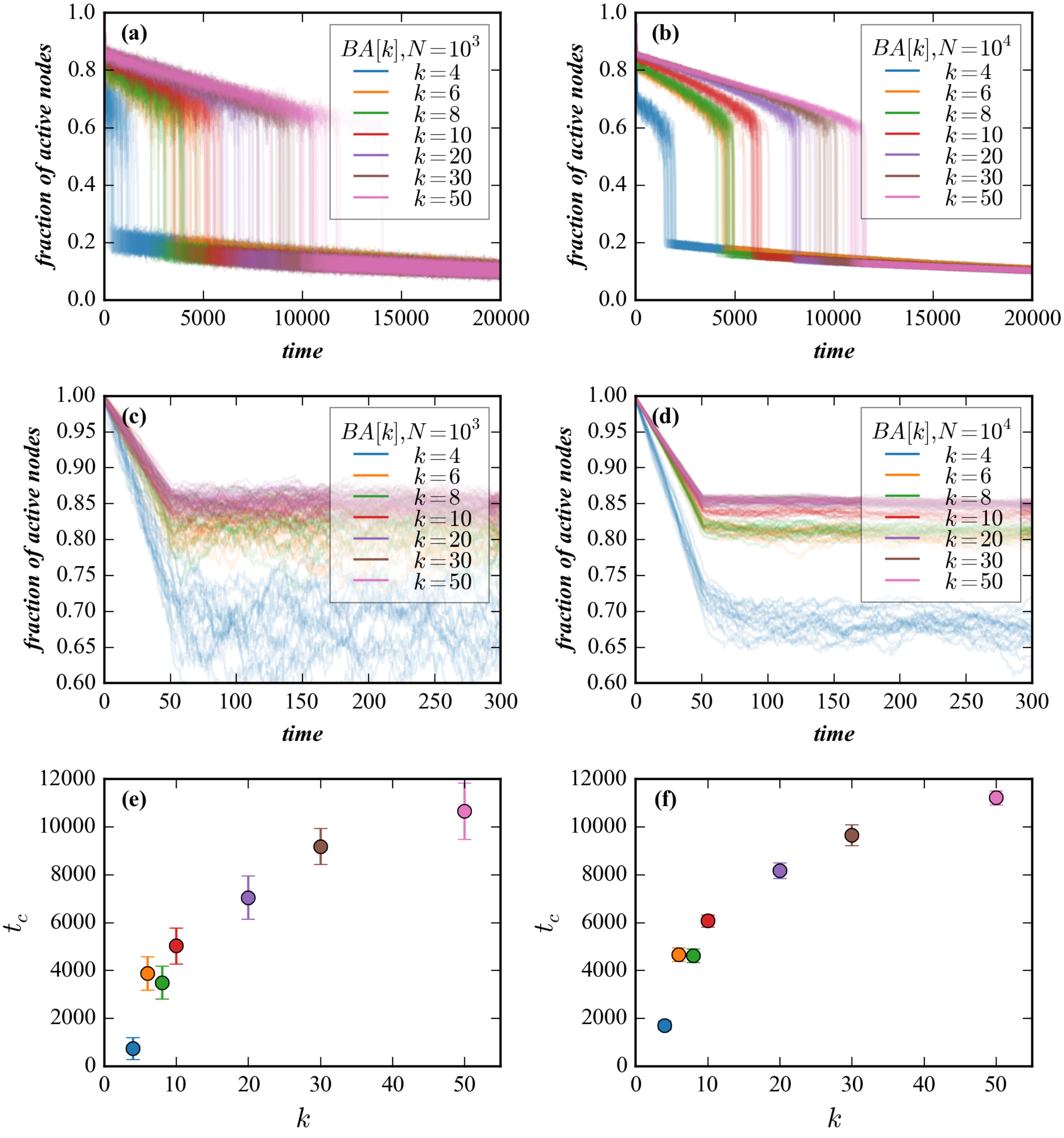

Figure S1. The dependence of t_c on the network size and average degree for BA random network. We show 20 realizations of fraction of active nodes f_a for each BA network with average degree k and with network size $N = 10^3$ (left) and $N = 10^4$ (right) for first (a,b) 20000 steps and (c,d) 300 steps. From these realizations we calculate mean and standard deviation of t_c for each BA network with average degree k (e,f). We use model with fixed parameters $p = 0.003$, $r = 0.8$, $q = 0.99$ and $T_h = 0.5$.

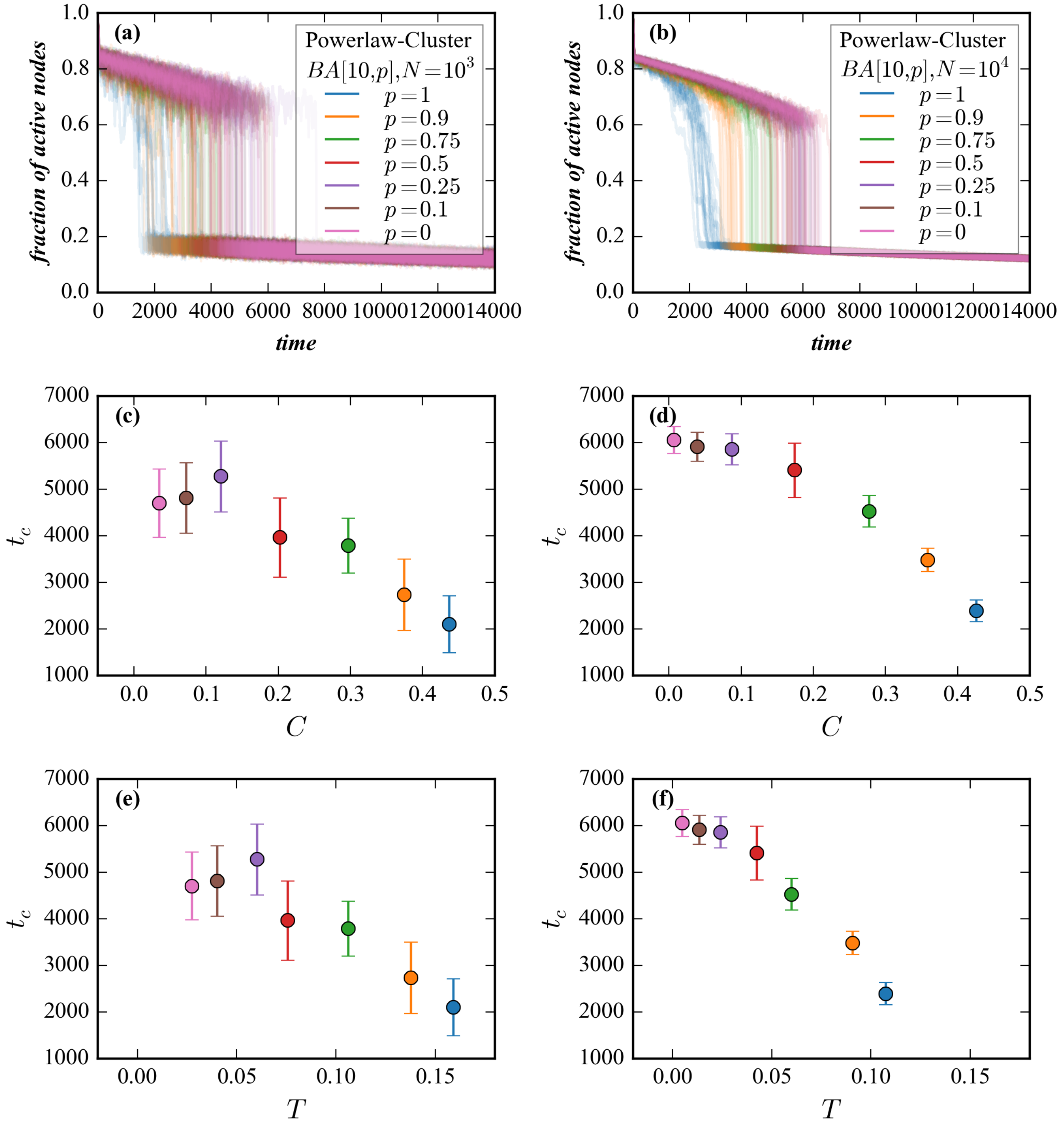

Figure S2. The dependence of t_c on the average clustering coefficient and transitivity for BA random networks with tunable clustering. Similarly as in Fig. S1, we show 20 realizations of fraction of active nodes f_a for each BA network with tuned clustering[1] with network size (a) $N = 10^3$ and (b) $N = 10^4$. From these realizations we calculate mean and standard deviation of t_c for each BA network with tuned clustering and show t_c dependence on (c,d) average clustering coefficient and (e,f) transitivity. We use model with fixed parameters $p = 0.003$, $r = 0.8$, $q = 0.99$ and $T_h = 0.5$.

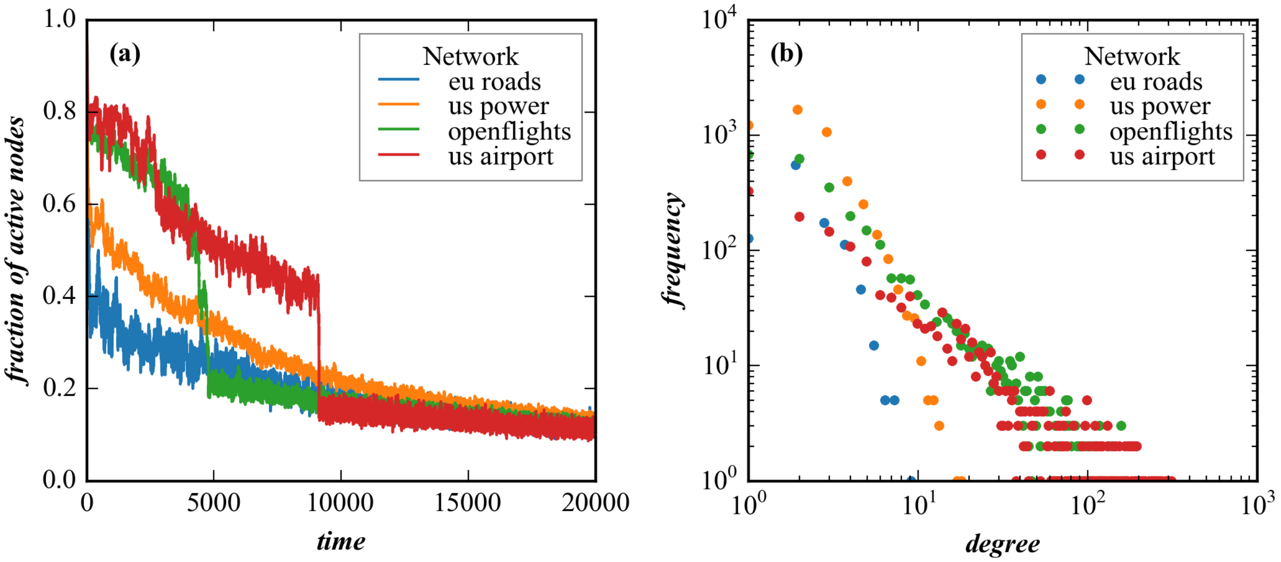

Figure S3. A realization of model dynamics on technical networks (I). Shown are (a) fraction of active nodes f_a as dynamics output of decaying model with fixed parameters $p = 0.003$, $r = 0.8$, $q = 0.99$ and $T_h = 0.5$ and (b) initial degree distribution, for physical infrastructure networks (US Power Grid,[2] Euro Road,[3] Open Flights[4,5] and US Airports[4,5]).

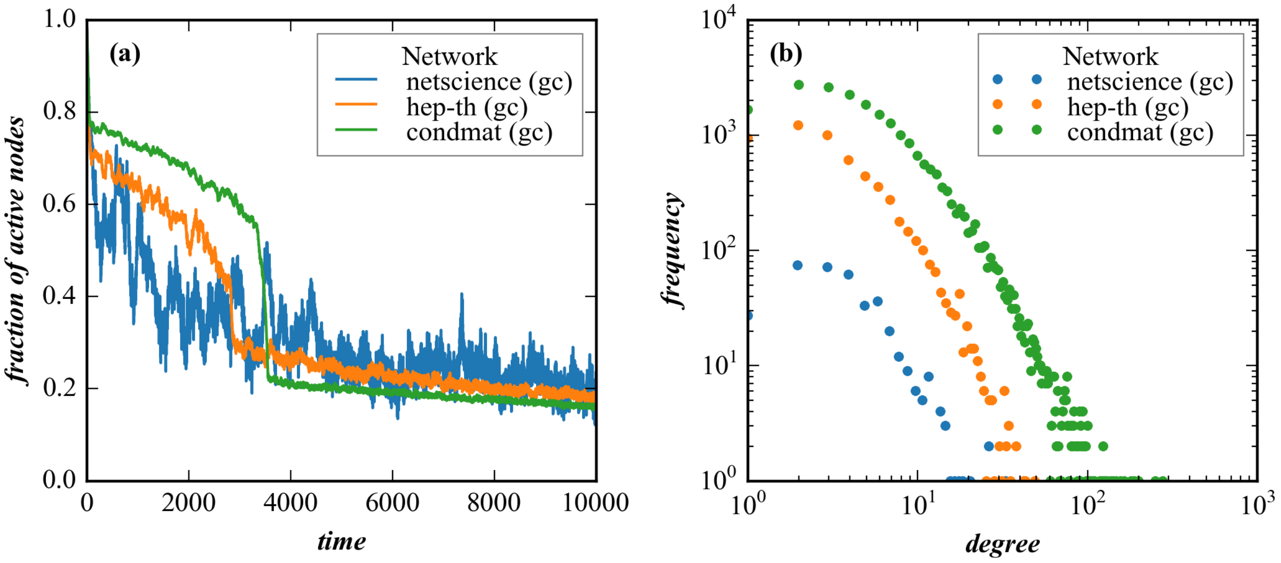

Figure S4. A realization of model dynamics on collaborative networks (giant components). Shown are (a) fraction of active nodes f_a as dynamics output of decaying model with fixed parameters $p = 0.003$, $r = 0.8$, $q = 0.99$ and $T_h = 0.5$ and (b) initial degree distribution, for giant components of scientific collaboration networks[6] (CondMat, HepTh, NetScience).

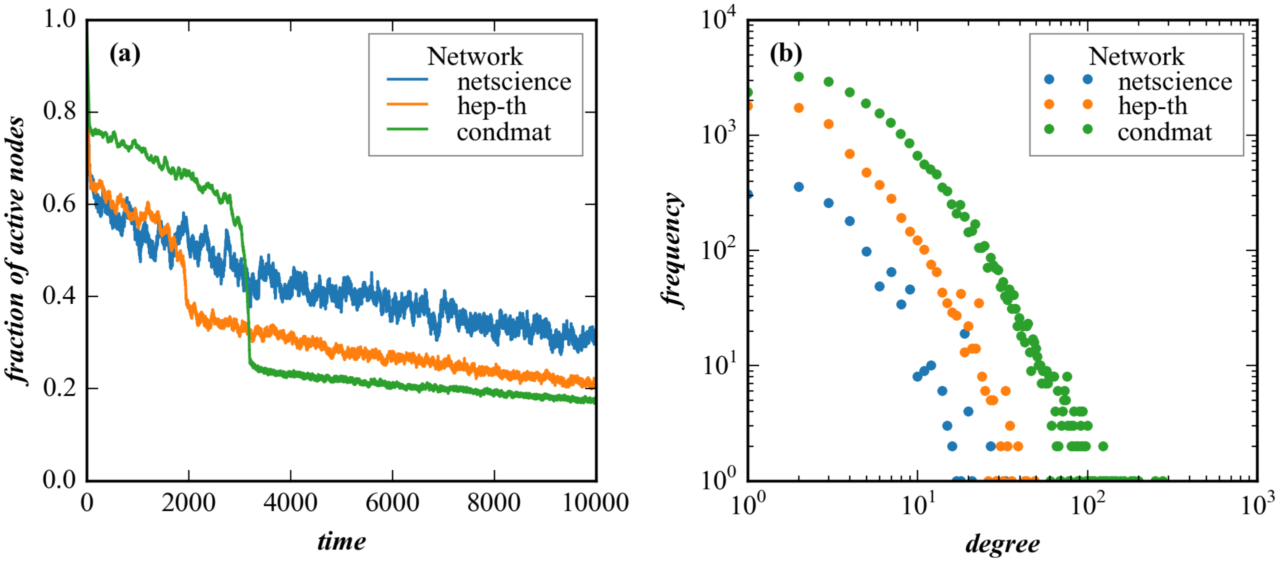

Figure S5. A realization of model dynamics on collaborative networks. Shown are (a) fraction of active nodes f_a as dynamics output of decaying model with fixed parameters $p = 0.003$, $r = 0.8$, $q = 0.99$ and $T_h = 0.5$ and (b) initial degree distribution, for scientific collaboration networks[6] (CondMat, HepTh, NetScience).

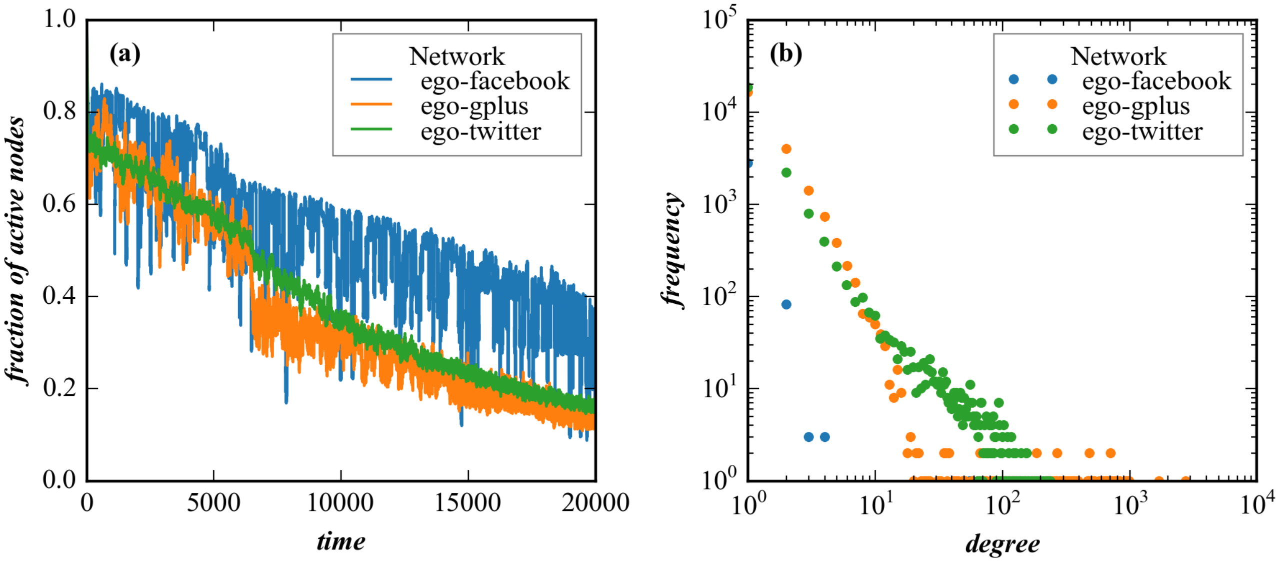

Figure S6. A realization of model dynamics on networks from social systems (I). Shown are (a) fraction of active nodes f_a as dynamics output of decaying model with fixed parameters $p = 0.003$, $r = 0.8$, $q = 0.99$ and $T_h = 0.5$ and (b) initial degree distribution, for interaction networks in online social networking platforms[7] (ego-Facebook, ego-Twitter, ego-gPlus).

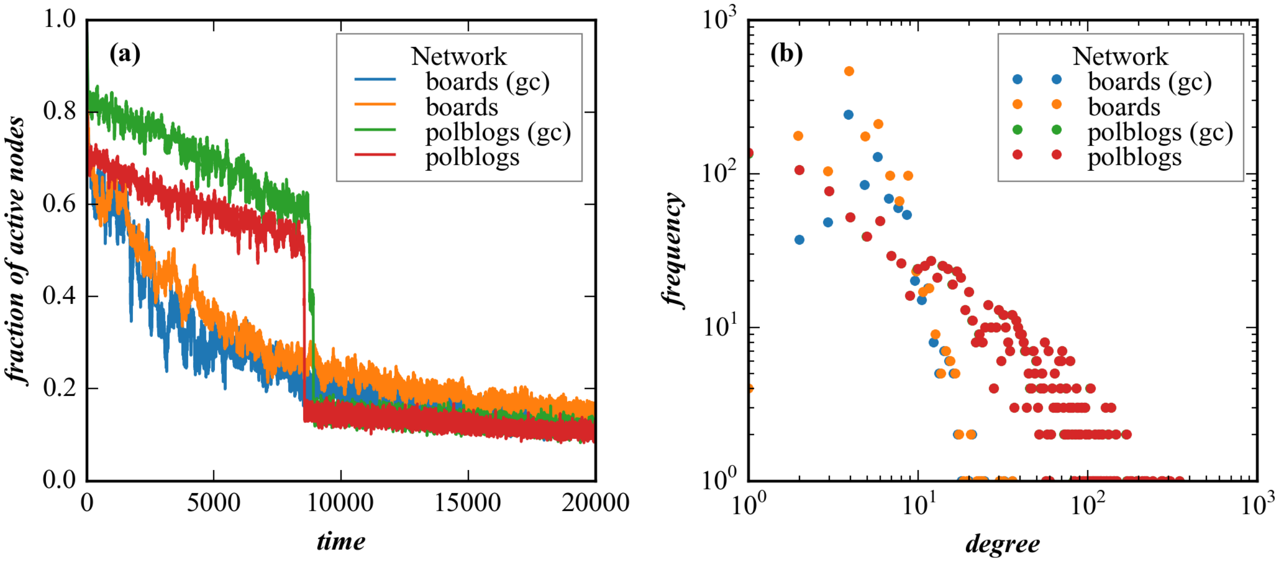

Figure S7. A realization of model dynamics on networks from social systems (II). Shown are (a) fraction of active nodes f_a as dynamics output of decaying model with fixed parameters $p = 0.003$, $r = 0.8$, $q = 0.99$ and $T_h = 0.5$ and (b) initial degree distribution, for network of political blogosphere (Blogs[8]) and network of connections between board of directors in public companies in Norway (Boards[9]), and their corresponding giant components (gc).

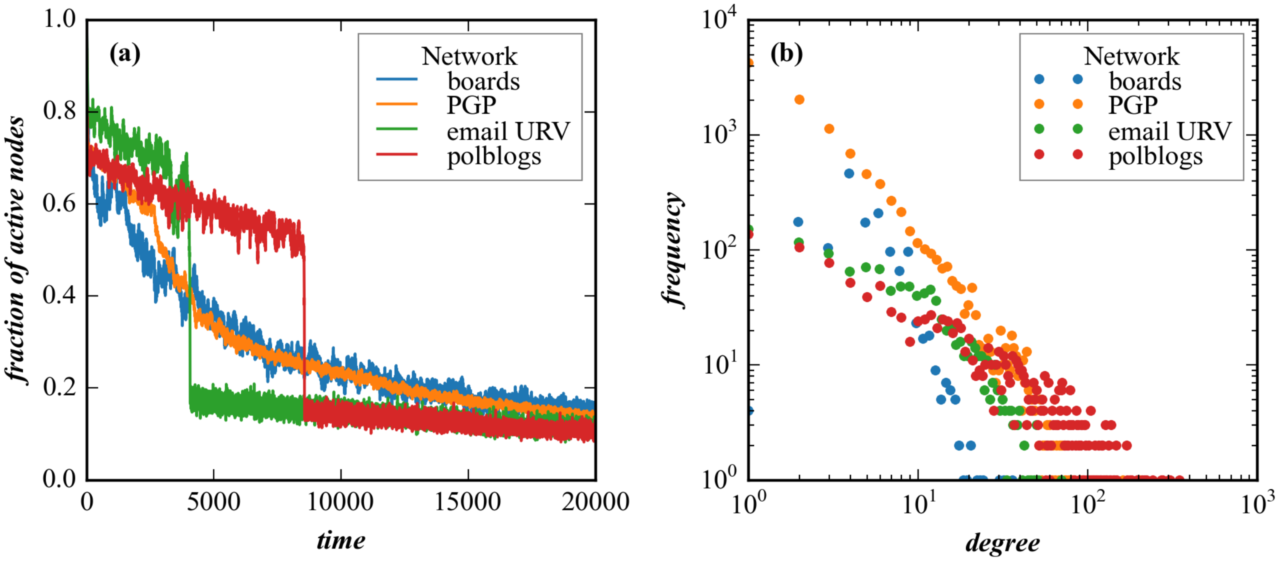

Figure S8. A realization of model dynamics on networks from social systems (III). Shown are (a) fraction of active nodes f_a as dynamics output of decaying model with fixed parameters $p = 0.003$, $r = 0.8$, $q = 0.99$ and $T_h = 0.5$ and (b) initial degree distribution, for personal communication networks (PGP[10] and email URV[11]), networks of political blogosphere (Blogs[8]) and connections between board of directors in public companies in Norway (Boards[9]).

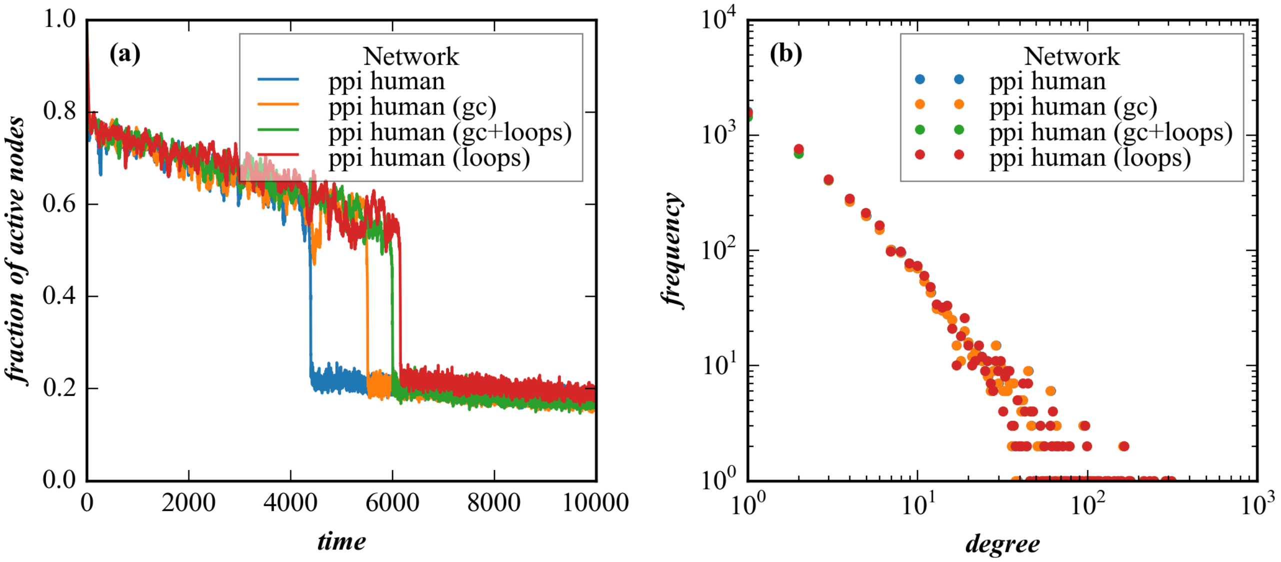

Figure S9. A realization of model dynamics on biological network. Shown are (a) fraction of active nodes f_a as dynamics output of decaying model with fixed parameters $p = 0.003$, $r = 0.8$, $q = 0.99$ and $T_h = 0.5$ and (b) initial degree distribution, for human interactome network[12] with and without self-loops and its giant component with and without self-loops.

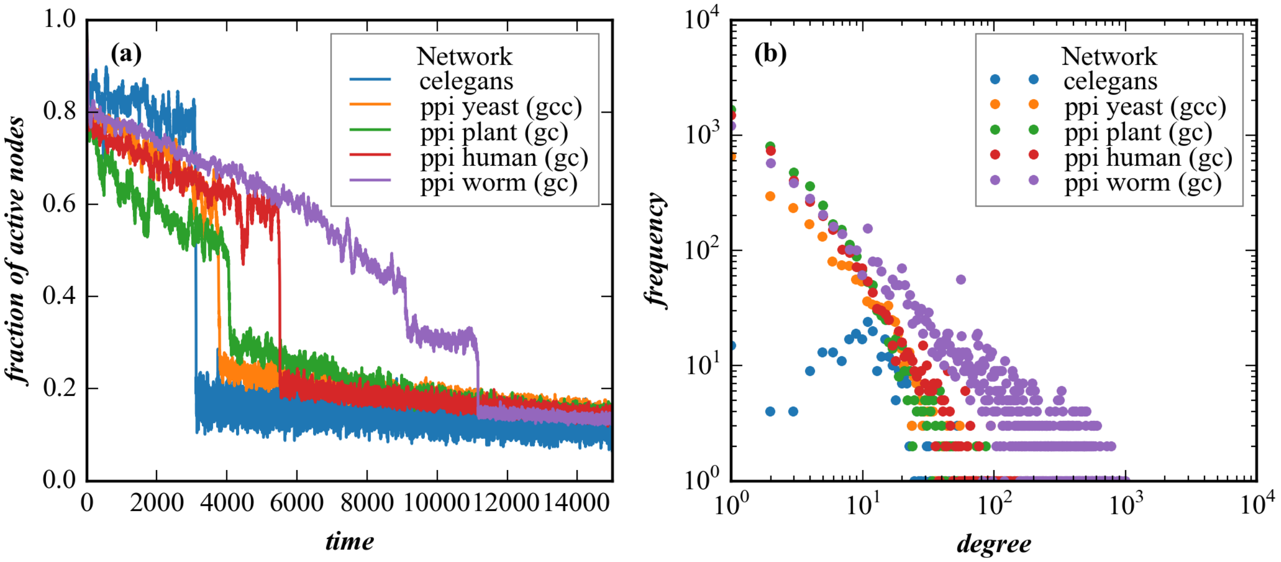

Figure S10. A realization of model dynamics on biological networks (giant components). Shown are (a) fraction of active nodes f_a as dynamics output of decaying model with fixed parameters $p = 0.003$, $r = 0.8$, $q = 0.99$ and $T_h = 0.5$ and (b) initial degree distribution, for the giant components of human,[12] plant,[13] worm[2] and yeast[14] interactome network.

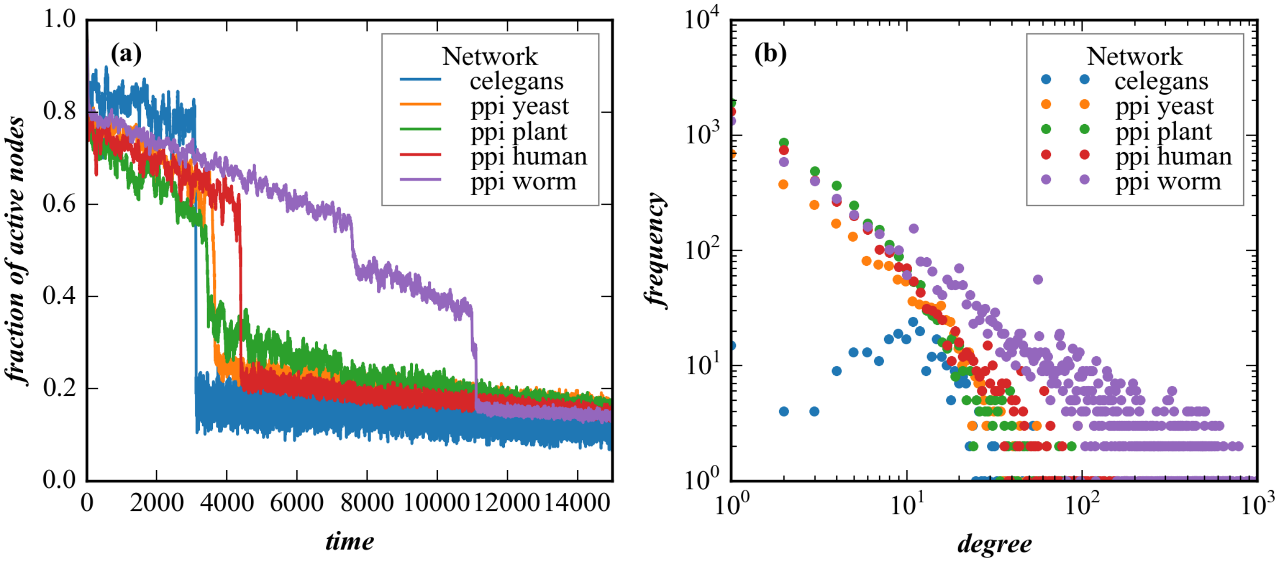

Figure S11. A realization of model dynamics on biological networks. Shown are (a) fraction of active nodes f_a as dynamics output of decaying model with fixed parameters $p = 0.003$, $r = 0.8$, $q = 0.99$ and $T_h = 0.5$ and (b) initial degree distribution, for the human,[12] plant,[13] worm[2] and yeast[14] interactome network.

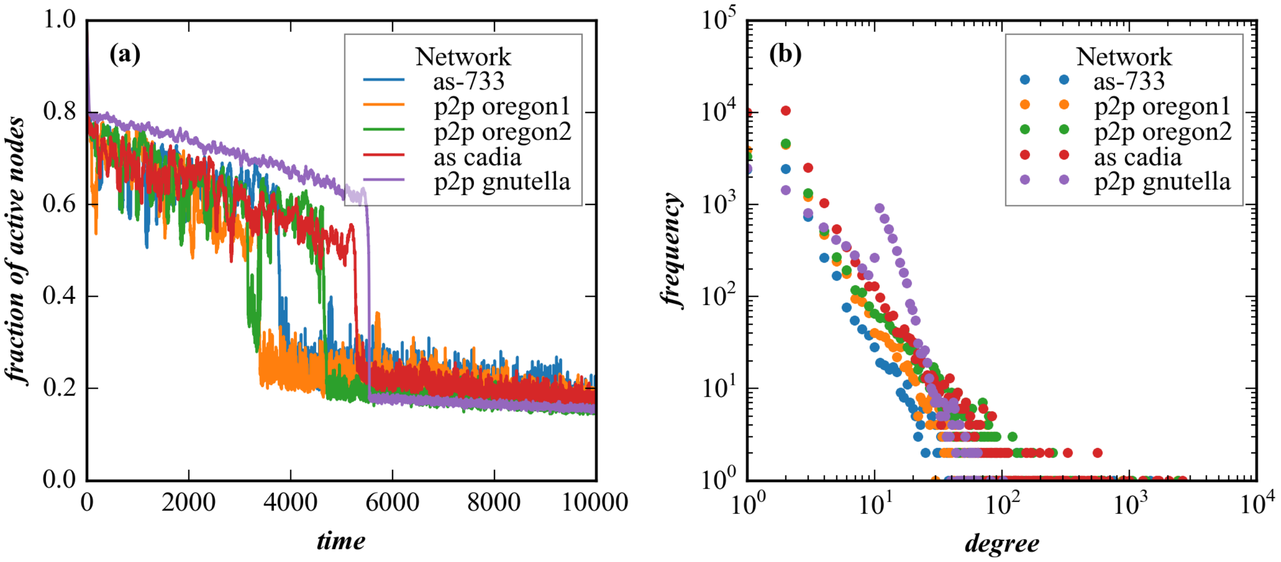

Figure S12. A realization of model dynamics on technical networks (II). Shown are (a) fraction of active nodes f_a as dynamics output of decaying model with fixed parameters $p = 0.003$, $r = 0.8$, $q = 0.99$ and $T_h = 0.5$ and (b) initial degree distribution, for computer networks[15] that include networks of autonomous systems of the Internet from CAIDA and Route Views projects (AS-733, Oregon1, Oregon2) together with peer-to-peer file sharing network Gnutella.

S2 Supplementary tables

network	source	N	L	$<k>$	$<k^2>$	$loops$	cc	gc_size	C	r	T	α	σ	x_{min}
us power	KONECT[16]	4941	6594	2.6691	3.2087	0	1	4941	0.0801	0.0035	0.1032	7.6292	0.9193	10
us airport	KONECT[16]	1574	17215	21.8742	1976.2917	0	2	1572	0.5042	-0.1133	0.3841	1.8529	0.0382	13
eu road	KONECT[16]	1039	1305	2.512	1.4433	0	1	1039	0.0189	0.09	0.0353	6.3583	0.6315	5
as cadia	KONECT[16]	26475	53381	4.0326	1113.8349	0	1	26475	0.2082	-0.1946	0.0073	2.0919	0.0244	6
as-733	KONECT[16]	6474	12572	3.8838	624.9948	0	1	6474	0.2522	-0.1818	0.0096	2.0668	0.0481	6
as-733 (loops)	KONECT[16]	6474	13895	4.2926	628.9104	1323	1	6474	0.2522	-0.1704	0.0096	2.1479	0.0565	8
openflights	KONECT[16]	2939	15677	10.6683	480.7504	0	11	2905	0.4526	0.0509	0.2547	1.7384	0.0155	2
openflights (gc)	KONECT[16]	2905	15645	10.7711	485.4364	0	1	2905	0.4555	0.0489	0.2547	1.7362	0.0155	2
p2p gnutella	SNAP[17]	10876	39994	7.3545	48.6485	0	1	10876	0.0062	-0.0132	0.0054	4.5833	0.0655	12
oregon1	SNAP[17]	11174	23409	4.1899	1095.2666	0	1	11174	0.2964	-0.1945	0.0096	2.0836	0.04	7
oregon2	SNAP[17]	11461	32730	5.7115	1239.7002	0	1	11461	0.3517	-0.1643	0.037	1.9447	0.0201	4
condmat	SNAP[17]	23133	93439	8.0784	112.9339	0	567	21363	0.6334	0.134	0.2643	3.4256	0.0741	27
condmat (gc)	SNAP[17]	21363	91286	8.5462	118.996	0	1	21363	0.6417	0.1253	0.2618	3.4256	0.0741	27
hep-th	Newman[18]	8361	15751	3.7677	18.5357	0	1332	5835	0.442	0.2939	0.3296	3.448	0.0937	10
hep-th (gc)	Newman[18]	5835	13815	4.7352	20.7671	0	1	5835	0.5062	0.1852	0.2841	5.2648	0.3351	18
netscience	Newman[18]	1589	2742	3.4512	12.0362	0	396	379	0.6378	0.4616	0.6934	3.6067	0.179	7
netscience (gc)	Newman[18]	379	914	4.8232	15.4226	0	1	379	0.7412	-0.0817	0.4306	3.3598	0.224	6
polblogs	Newman[18]	1490	16718	22.4403	1319.8706	3	268	1222	0.2627	-0.2212	0.226	3.6673	0.2543	79
polblogs (gc)	Newman[18]	1222	16717	27.3601	1474.764	3	1	1222	0.3203	-0.2213	0.226	3.6673	0.2543	79
boards	Opshal[19]	1494	4065	5.4418	10.0512	0	126	818	0.9185	0.1666	0.6805	4.3723	0.3794	11
boards (gc)	Opshal[19]	818	2577	6.3007	13.467	0	1	818	0.8752	0.0188	0.5965	4.1211	0.2154	8
PGP	KONECT[16]	10680	24316	4.5536	65.2413	0	1	10680	0.2659	0.2382	0.378	4.2652	0.255	36
email URV	KONECT[16]	1133	5451	9.6222	87.2289	0	1	1133	0.2202	0.0782	0.1663	6.7745	1.0208	35
ego-facebook	KONECT[16]	2888	2981	2.0644	523.8698	0	1	2888	0.0272	-0.6682	0.0004	4.5239	0.0656	1
ego-twitter	KONECT[16]	23370	32831	2.8097	100.2793	0	72	22322	0.0692	-0.478	0.0215	1.889	0.0172	3
ego-gplus	KONECT[16]	23628	39194	3.3176	1239.8777	0	4	23613	0.1741	-0.3885	0.0037	2.6223	0.019	2
ppi human	CCSB[16]	4303	13427	6.2408	251.5456	0	135	4100	0.052	-0.2135	0.0333	2.1952	0.0432	8
ppi human (loops)	CCSB[20]	4303	13944	6.4811	256.4932	517	135	4100	0.052	-0.2079	0.0333	2.2056	0.0425	8
ppi human (gc)	CCSB[20]	4100	13358	6.5161	262.378	0	1	4100	0.0546	-0.2165	0.0333	2.1952	0.0432	8
ppi human (gc+loops)	CCSB[20]	4100	13797	6.7302	267.8604	439	1	4100	0.0546	-0.2124	0.0333	2.2056	0.0425	8
ppi plant	CCSB[20]	4866	10928	4.4916	97.4686	0	161	4519	0.0982	-0.1905	0.0374	2.5961	0.0625	8
ppi plant (gc)	CCSB[20]	4519	10721	4.7449	104.0028	0	1	4519	0.0986	-0.1974	0.0372	2.5961	0.0625	8
ppi worm	CCSB[20]	6176	177267	57.4051	15280.0979	0	118	5966	0.3575	0.1527	0.4719	5.8312	0.4692	528
ppi worm (gc)	CCSB[20]	5966	177161	59.3902	15702.0324	0	1	5966	0.3667	0.1517	0.4719	5.8312	0.4692	528
ppi yeast	CCSB[20]	2361	7182	6.0839	65.2602	536	101	2224	0.1301	-0.0846	0.1023	3.3387	0.1421	15
ppi yeast (gc)	CCSB[20]	2224	7049	6.339	68.109	440	1	2224	0.1381	-0.0977	0.1023	3.3387	0.1421	15
celegans	Newman[18]	297	2148	14.4646	167.5551	0	1	297	0.2924	-0.1632	0.1807	3.3427	0.2234	15

Table S1. List of the real networks used in the study with their corresponding structural properties and data sources. From left to right, we report: network name, data source where the network was retrieved, number of nodes, number of links, mean degree, degree variance, number of loops, number of connected components, size of the giant component, clustering coefficient, transitivity, estimated power law exponent, standard error of the power law exponent and minimal degree for the estimated power law exponent.

network	N	$<k>$	$\bar{t_c}$	σ_{t_c}	$\bar{t_0}$	σ_{t_0}	$\bar{t_1}$	σ_{t_1}	$\bar{d}_{t_1-t_0}$	$\sigma_{t_1-t_0}$
BA	1000	2	4167.95	874	7763.7	878.2444	13587.2	1082.0206	5823.5	1150.0873
BA	1000	3	1597.2	551.5929	2670.8	569.2061	3081.4	680.0708	410.6	528.2811
BA	1000	4	733	455.3478	743.1	446.8958	774.65	447.5457	31.55	9.4338
BA	1000	6	3877.7	701.0481	3966	595.7928	3981.45	595.2144	15.45	3.2521
BA	1000	8	3495.1	683.8985	3492.4	683.0756	3502.55	683.2398	10.15	1.4965
BA	1000	10	5026.95	744.415	5024.9	744.354	5033.2	743.7541	8.3	1.1286
BA	1000	20	7041.35	905.1482	7039.7	904.7559	7044.5	905.1318	4.8	0.7678
BA	1000	30	9184.5	754.6323	9182.7	754.4474	9187.2	754.6166	4.5	0.8885
BA	1000	50	10665.5	1179.4073	10663.8	1178.9213	10667.65	1179.2533	3.85	0.8751
BA	10000	2	5776.55	444.4148	6070.7	291.332	16560.7	360.1647	10490	365.9577
BA	10000	3	2937.55	279.3917	2933.1	283.4695	5795.4	355.628	2862.3	324.8827
BA	10000	4	1710.1	191.6806	1688.1	193.0429	1738.75	188.7546	50.65	13.808
BA	10000	6	4668.35	281.5875	4662.3	281.087	4684.05	281.396	21.75	4.8545
BA	10000	8	4623.65	282.8963	4617.9	282.9225	4631.15	282.4713	13.25	1.4824
BA	10000	10	6071.8	257.7813	6068.05	257.2747	6078.3	257.74	10.25	1.1642
BA	10000	20	8174.4	330.8895	8172.1	330.6524	8177.95	331.014	5.85	0.6708
BA	10000	30	9653.15	437.7822	9651	437.4512	9655.9	437.6804	4.9	0.6407
BA	10000	50	11216.35	287.657	11214.4	287.3469	11218.65	287.5626	4.25	0.5501
celegans	297	14.46465	3285.65	1166.7327	3308.2	1160.4045	3321.4	1158.8053	13.2	5.917
netscience (gc)	379	4.82322	438.15	261.8528	2867.2	870.5796	4151.85	1145.9483	1284.65	1519.9385
boards (gc)	818	6.30073	1244.45	339.2229	2679.4	561.5451	6028.5	871.5187	3349.1	875.1788
eu road	1039	2.51203	78.95	10.6003	103.05	89.1973	3808.95	888.5526	3705.9	833.7455
email URV	1133	9.62224	3782.5	755.8097	3778.65	755.3197	3806.05	747.4566	27.4	15.6252
polblogs (gc)	1222	27.36007	8128.8	1040.0312	8124.95	1038.9876	8353.4	953.9667	228.45	263.2494
polblogs	1490	22.44027	7984.8	1156.1224	7966.3	1151.8015	8084.05	1136.5556	117.75	136.7121
boards	1494	5.44177	1475.6	307.0838	2643.45	466.9653	10215.65	706.5427	7572.2	829.206
us airport	1574	21.87421	5659	1311.4	8102.2	1252.9475	9207.2	1643.8479	1105	775.5937
netscience	1589	3.45123	1861.65	409.3133	3322.25	522.3633	13716.65	937.2261	10394.4	980.2842
ppi yeast (gc)	2224	6.33903	3964.35	476.0425	3968.35	474.44	4159.4	458.4026	191.05	181.8723
ppi yeast	2361	6.08386	4030.7	652.4993	4025.25	651.5496	6251.5	632.3966	2226.25	866.5091
ego-facebook	2888	2.0644	1015.15	1329.3599	12085.45	4989.4379	10989.85	4583.0542	-1095.6	4109.177
openflights (gc)	2905	10.77108	4601.1	1065.0202	4894.1	832.3168	5381.8	906.7952	487.7	519.7974
openflights	2939	10.66825	4690.4	982.0117	4975.3	873.7642	5500.35	1058.4153	525.05	476.8668
ppi human (gc)	4100	6.5161	5706.9	939.3882	5721.45	954.2952	5758.45	954.6529	37	14.9772
ppi human (gc+loops)	4100	6.73024	6379.15	1269.0769	6374.85	1268.7357	6415	1256.7436	40.15	30.8447
ppi human	4303	6.24076	5747.35	972.3058	5726.25	962.1919	5789.7	947.0449	63.45	46.1673
ppi human (loops)	4303	6.48106	5829.55	1037.9046	5820	1034.7066	6134.4	786.3613	314.4	430.193
ppi plant (gc)	4519	4.74485	3394	539.5275	3511	480.7938	6775.05	695.5357	3264.05	558.1414
ppi plant	4866	4.49157	3312.65	547.3055	3462.15	650.3395	8090.65	611.262	4628.5	748.5327
us power	4941	2.6691	1039.1	185.7318	1810.9	250.5581	10103.35	488.271	8292.45	446.2023
hep-th (gc)	5835	4.73522	2181.7	339.4911	2207.4	342.5229	6965.45	464.3239	4758.05	608.3649
ppi worm (gcc)	5966	59.39021	7509.1	493.8995	7884.25	485.4481	10144.35	809.3067	2260.1	803.0821
ppi worm	6176	57.40512	7632.85	575.5288	7820.7	613.1589	9917.55	924.2904	2096.85	826.7018
as-733	6474	3.88384	1901.65	1048.6385	3085.8	1261.4212	4656.2	583.5296	1570.4	1107.2402
as-733 (loops)	6474	4.29255	2954.85	1215.093	3573.25	1098.3557	5759.8	608.1704	2186.55	1190.6144
hep-th	8361	3.76773	2072	250.419	2050.35	268.2001	10635.1	406.8033	8584.75	394.9977
PGP	10680	4.55356	2738.4	252.2141	2807	222.6628	10705.3	337.6863	7898.3	372.934
p2p gnutella	10876	7.35454	5462.2	183.74	5455.85	183.4578	5471.5	183.7256	15.65	2.1831
oregon1	11174	4.18991	2318.95	858.4213	3294.95	798.1081	3840	548.3	545.05	414.9338
oregon2	11461	5.71154	2516.95	995.7826	3497.1	989.5173	4596.95	624.8323	1099.85	960.5316
condmat (gc)	21363	8.54618	3196	127.4412	3175.6	131.548	4045.3	186.1788	869.7	192.0765
condmat	23133	8.07842	3286.45	238.2189	3265.15	237.0091	6197.8	177.2645	2932.65	284.9916
ego-twitter	23370	2.80967	7462.4	466.088	6805.25	413.5035	15320.2	461.1628	8514.95	511.3261
ego-gplus	23628	3.31759	4753.15	1596.9852	6968.8	989.0266	11984.2	1127.0694	5015.4	1565.5948
as cadia	26475	4.03256	4428.9	957.0195	4552.35	824.2417	5568.95	443.2308	1016.6	648.0813

Table S2. Analysis of t_c for real networks. From left to right, we report: network name, number of nodes, mean degree, mean t_c, standard deviation of t_c, mean and standard deviation of the start of crash period (after that time point, the fraction of active nodes is below threshold), mean and standard deviation of the end of crash period (after that time point, the fraction of active nodes decreased below $1-r$) and finally mean and standard deviation for the difference between end and start crash time. We use 20 realizations of model with $p = 0.003$, $q = 0.99$, $r = 0.8$, and $T_h = 0.5$.

References

1. Holme, P. & Kim, B. J. Growing scale-free networks with tunable clustering. *Phys. Rev. E* **65**, 026107 (2002).
2. Watts, D. J. & Strogatz, S. H. Collective dynamics of 'small-world' networks. *Nature* **393**, 440–442 (1998).
3. Šubelj, L. & Bajec, M. Robust network community detection using balanced propagation. *Eur. Phys. J. B* **81**, 353–362 (2011).
4. Opsahl, T., Agneessens, F. & Skvoretz, J. Node centrality in weighted networks: Generalizing degree and shortest paths. *Soc. Networks* **32**, 245–251 (2010).
5. Opsahl, T. Why anchorage is not (that) important: Binary ties and sample selection. Website (2011). Available at: http://toreopsahl.com/2011/08/12/why-anchorage-is-not-that-important-binary-tiesand-sample-selection. (Accessed: 30th May 2015).
6. Newman, M. E. The structure of scientific collaboration networks. *Proc. Natl. Acad. Sci. USA* **98**, 404–409 (2001).
7. Leskovec, J. & Mcauley, J. J. Learning to discover social circles in ego networks. *Adv. Neural Inf. Process. Syst.* **25**, 539–547 (2012).
8. Adamic, L. A. & Glance, N. The political blogosphere and the 2004 u.s. election: Divided they blog. Paper presented at the 3rd international workshop on Link discovery, 36–43. New York, NY, USA: ACM Press. (10.1145/1134271.1134277)(2005).
9. Seierstad, C. & Opsahl, T. For the few not the many? the effects of affirmative action on presence, prominence, and social capital of women directors in norway. *Scand. J. Manag.* **27**, 44–54 (2011).
10. Boguñá, M., Pastor-Satorras, R., Díaz-Guilera, A. & Arenas, A. Models of social networks based on social distance attachment. *Phys. Rev. E* **70**, 056122 (2004).
11. Guimerŕ, R., Danon, L., Díaz-Guilera, A., Giralt, F. & Arenas, A. Self-similar community structure in a network of human interactions. *Phys. Rev. E* **68**, 065103 (2003).
12. Rolland, T. *et al.* A proteome-scale map of the human interactome network. *Cell* **159**, 1212–1226 (2014).
13. Dreze, M. *et al.* Evidence for network evolution in an arabidopsis interactome map. *Science* **333**, 601–607 (2011).
14. Yu, H. *et al.* High-quality binary protein interaction map of the yeast interactome network. *Science* **322**, 104–110 (2008).
15. Leskovec, J., Kleinberg, J. & Faloutsos, C. Graph evolution: Densification and shrinking diameters. *ACM Trans. Knowl. Discov. Data* **1**, 2 (2007).
16. Kunegis, J. Konect: the koblenz network collection. In *Proceedings of the 22nd international conference on World Wide Web companion*, 1343–1350 (International World Wide Web Conferences Steering Committee, Switzerland, 2013).
17. Leskovec, J. & Krevl, A. SNAP Datasets: Stanford large network dataset collection. Website (2014). Available at: http://snap.stanford.edu/data. (Accessed: 30th May 2015).
18. Newman, M. Mark Newman: Network data. Website (2013). Available at: http://www-personal.umich.edu/~mejn/netdata/. (Accessed: 30th May 2015).
19. Opsahl, T. Tore Opsahl: Datasets. Website (2011). Available at: http://toreopsahl.com/datasets/. (Accessed: 30th May 2015).
20. for Cancer Systems Biology, C. C. Ccsb interactome database. Website (2015). Available at: http://interactome.dfci.harvard.edu/. (Accessed: 30th May 2015).
